# Acute toxicity of copper to *Galaxea fascicularis* and its utility as a candidate standard coral toxicity test species

**DOI:** 10.1016/j.toxrep.2026.102363

**Published:** 2026-10-28

**Authors:** Annaleise J. Conway, Andrew Heyes, Carys L. Mitchelmore

**Affiliations:** University of Maryland Center for Environmental Science, Chesapeake Biological Laboratory, 146 Williams Street, Solomons, MD 20688, USA

**Keywords:** Copper, Mortality, Metal toxicity, Bleaching, Coral, Galaxea fascicularis

## Abstract

Corals face exposure to numerous chemical contaminants, yet toxicity data is limited even for common coastal contaminants. While representative standard toxicity test organisms are typically used to assess environmental risk, these may not be good surrogates for species with unique attributes, including coral symbiotic relationships. Because hard corals are ecologically important yet challenging to obtain and maintain under laboratory conditions, we evaluated the utility of *Galaxea fascicularis,* a common and globally available aquaculture species. Methods were adapted from OECD and US EPA guidelines for acute invertebrate toxicity testing, with modifications to ensure organism health. Copper was selected given its common occurrence in coastal ecosystems and use as a positive toxicity test control. Three copper acute (96 h) daily renewal tests were conducted with analytical verification of exposure concentrations. Mortality was the primary endpoint due to its use in risk assessment; however additional endpoints including polyp retraction, bleaching and symbiont health (measured via light-adapted photosynthetic efficiency) were also evaluated. The three independent tests yielded reproducible and reliable results, establishing a 96 h median lethal concentration (LC50) of 468 ± 70 µg/L dissolved copper. These findings demonstrate that *G. fascicularis* is a promising candidate standard global toxicity test species for hard corals and highlights the potential utility of incorporating more sensitive and reproducible coral-specific biological endpoints. Additional research, including acute and chronic testing with additional common coastal contaminants and inter-laboratory testing are required to compare the sensitivity of this species to other coral to establish this species as a standard coral test organism.

## Highlights

• *Galaxea fascicularis* is a promising candidate species for standardized coral toxicity testing.
• EPA and OECD guidelines were used to develop modified standard toxicity test methods for corals.
• An average LC50 of 468 (± 70) µg/L dissolved copper was observed.
• Polyp retraction was a highly sensitive and well replicated biological endpoint.
• Sensitivity of *Galaxia fascicularis* compared to other hard corals requires testing with other chemicals.

## Graphical Abstract

**Figure:**
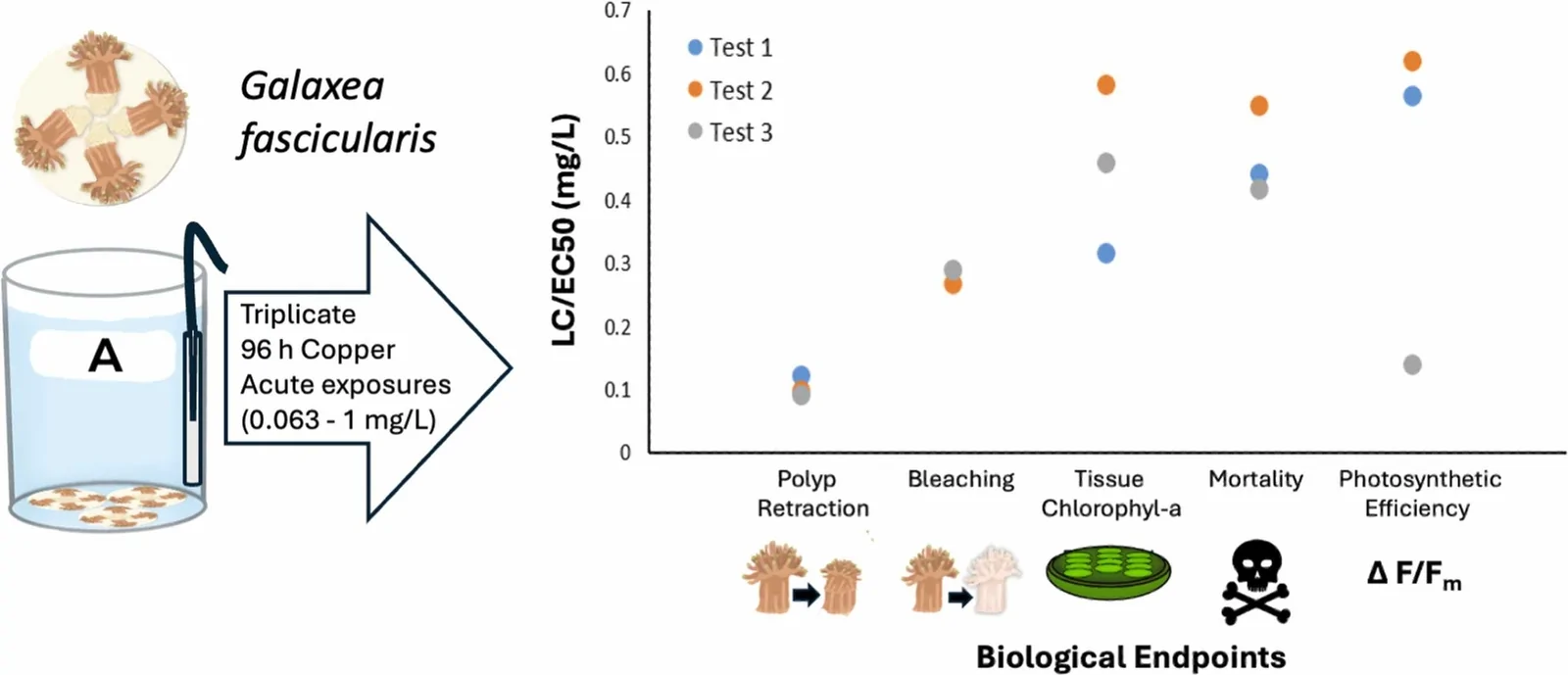

## 1 Introduction

Coral reefs are among the most economically and biologically valuable ecosystems in the world [1]. However, they are being negatively impacted by a combination of stressors, including rising sea surface temperatures and other climate change ramifications, diseases, and pollution [2], [3]. Estimates indicate that 60% of global reefs are threatened by natural and anthropogenic stressors [4], [5], including chemical contaminants with approximately 14% of the world’s coral lost between 2009 and 2018 [6]. Regulatory bodies such as the U.S. Environmental Protection Agency (EPA) or Organization for Economic Co-operation and Development (OECD) have established standardized frameworks to assess chemical toxicity. These frameworks consist of standardized testing protocols using a limited number of surrogate species meant to represent broader taxonomic groups. Therefore, a limited number of test organisms are available to cover global biodiversity, a problem particularly of concern for tropical marine species like scleractinian corals. Standard test organisms need to be widely available, amenable to laboratory culture conditions, and provide reliable and reproducible results [7]. Recently, Burns and Davies [8], Raimondo [9], and Raimondo et al. [10] highlighted how limitations in data reliability and quality in coral UV filter toxicity studies reduce their suitability for environmental risk assessments. Because of the need for reliable testing, suitable test species may not always represent the most sensitive organisms (e.g. [11]), but this tradeoff is necessary to make these testing protocols widely available.

Corals are unique symbiotic organisms that do not fit neatly into standard toxicity test organism categories. Even if suitable algae and invertebrate tests are carried out, they likely do not account for the interdependency of the intricate host-symbiont system. Currently, the closest standard US EPA and OECD test species is the marine invertebrate, mysid shrimp (*Americamysis bahia*), a tropical crustacean. However, cnidarians especially corals, differ fundamentally from crustaceans in reproduction, calcification, growth (especially in reef-building species), their sessile adult phase, as well as impacts to the algal symbiont that do not affect the coral host (e.g., phytotoxins such as herbicides). While studies have proposed the anemone *Aiptasia pulchella* as a model organism [12], [13], [14], evaluating reef-building corals directly is critical due to their importance as keystone species as well as their fragility in the current environment due to a variety of biological, chemical and physical stressors and potential differences as a calcifying species.

The need for standard coral tests and reliable and defensible data has been highlighted with the recent concerns over sunscreens and ecological risk (e.g., [8], [9], [10], [15]). A review by the National Academy of Sciences, Engineering and Medicine [16] recommended funding research on UV filter toxicity to support Ecological Risk Assessments (ERAs) for all UV filters currently marketed in the USA. The report emphasized that future studies should adhere to national/international standards where applicable, which may include new standards for toxicity testing on relevant species and the incorporation of non-traditional endpoints for acute and chronic toxicity testing [16]. Many reviews have criticized the lack of standard methods for coral toxicity testing hindering comparisons across studies and conclusions regarding a chemical toxicity and /or coral species/life stage sensitivity given the diversity of methods used across studies [17], [18]. Recent efforts have been directed towards establishing standardized bioassays in corals [19].

The hard coral *Galaxea fascicularis* (Linnaeus, 1767), a shallow-water species native to the Red Sea and Indo-Pacific region characterized by its large, green-tipped polyps [20] and possesses several traits ideal for a standard test organism. Globally sold in the aquarium trade due to its ease of culture in artificial seawater, *G. fascicularis* continually produces new polyps via budding. This provides a reliable and abundant supply of test organisms without requiring wild harvest or laboratory spawning allowing testing even at remote laboratories and a reduction in the environmental impact of testing. Furthermore, its large polyps (up to 10 mm in diameter) provides easy observations of individual polyps making assessment of mortality and polyp retraction straightforward without dissecting microscopes or macro lenses, unlike species with small polyps (2–3 mm) such as *Acropora* spp. Veron [21]. To evaluate *G. fascicularis* as a standard toxicity test organism, copper was selected as the representative toxicant. Copper is a common coastal contaminant with higher toxicity to corals compared with other metals [22] and serves as a standard reference toxicant [23], [24]. Additionally, the impact of copper on corals have been conducted to allow for cross-species comparisons, although to date the majority of studies have employed coral early life stages, with very few providing adult coral toxicity thresholds (e.g. [25], [26], [27], [28], [29], [30], [31], [32], [33], [34], [35], [36], [37], [38], [39], [40], [41], [42], [43], [44], [45], [46], [47]). Data regarding chemical impacts on *G. fascicularis* is extremely limited, although Sabdono [47] evaluated its 96 h acute mortality to copper.

In this study, we adapted OECD and US EPA acute toxicity testing guidelines for aquatic invertebrates and vertebrates [48], [49], [50] to establish an acute 96 h static-renewal testing protocol for *G. fascicularis*. The test methods employed were primarily based off of the EPA Mysid Acute Toxicity Test [50] with modifications to lighting scheme, salinity, and inclusion of additional biological endpoints to accommodate the needs of this particular species (Table 1). US EPA and OECD general guidelines for toxicity testing were also taken into account [48], [49]. In addition to mortality (median lethal concentration, LC50), we evaluated additional sublethal coral-specific biological endpoints including polyp retraction, plus a number of bleaching-related endpoints and algae symbiont health via pigment and photosynthetic efficiency measurements to provide median effect concentrations (EC50). These non-traditional endpoints were included as they have been commonly reported in coral toxicity studies and may be more sensitive and reliable markers of toxicological impact although their relationship to population level repercussions would need to be further explored [17]. Because toxicological sensitivity can be highly variable in corals [51], establishing standard benchmark data for *G. fascicularis* is essential to compare to other species so that appropriate adjustments can to be made in order to protect more sensitive taxa.

**Table 1:** Summary of acute testing guidelines from EPA and OECD aquatic studies. Summary of test conditions from this study are included for comparison. S = static, SS = semi-static (static renewal), FT = flow-through, SpD = species-dependent, NR = not reported, NA = not applicable, SD = standard deviation, Imm. = Immobilization, DT = during test, LC50 = median lethal concentration, EC50 = median effective concentration, IC50 = median inhibition concentration.

Guideline	OECD 203	OECD 202	OECD 201	OECD 235	OECD 236	OCSPP 850.1025	OCSPP 850.1035	OCSPP 850.1045	OCSPP 850.1055	OCSPP 850.1075	OCSPP 850.4500	This Study
Test Organism	Fish (various)	*Daphnia sp.*	Alga, Cyano-bacteria (various)	*Chironomus sp.*	Fish (various, embryo)	*C. virginica*	*A. bahia*	Penaeid Shrimp (various)	Bivalve (Embryo-Larval, various)	Fish (various)	Alga (various)	*G. fascicularis*
Test type	S, SS or FT	S	S	S	S or SS	FT	SS or FT	SS or FT	S	S, SS or FT	S	SS
Duration (h)	96	48	72	48	96	96	96	96	48	96	96	96
Temp C (± DT)	SpD (± 2)	18–22 (± 1)	21–24 (± 2)	SD (± 1)	26 (± 1)	20 (± 2)	25 (± 1)	23 (± 1)	SD (± 1)	SD (± 2)	SD (± 2)	25 (± 1)
Light intensity (lux)	540–1000	NR	SD	500–1000	540–1080	540–1080	540–1080	540–1080	540–1080	540–1080	60 µmol/m^2^/s	89 – 210 µmol/m^2^/s
Photoperiod (h light)	12–16	16^	SD	16^	12–16	12–16	12–16^&^	12–16^&^	12–16	12–16	14–24	12
Salinity (ppt; ± DT)	SpD	NA	NA	NA	NA	20 (± 2)	20 (± 2)	20 (± 2)	20 (± 2)	SD (± 2)	30 (± 5)	33 (± 2)
pH (± DT)	6.0–8.5	6–9 (± 1.5)	SD (± 1.5)	6–9 (± 1.5)	6.5–8.5 (± 1.5)	7.5–8.5	7.5–8.5 (± 1)	7.5–8.5 (± 1)	7.5–8.5 (± 1)	7.5–8.5 (± 1)	NA	8.0 (± 0.2 SD)
Organism/conc.	7	20	SpD	20	20	20	20	20	15–30 mL	7^@^	10000 cells/mL	12 polyps
Min. Replicates	1	4	3	4	1/well	2	2	2	2	1^@^	4	4
Exposure Concentrations	≥ 5 geometric series not exceeding 2.2	≥ 5 geometric series not exceeding 2.2	≥ 5 geometric series not exceeding 3.2	≥ 5 geometric series not exceeding 2.2	≥ 5 geometric series not exceeding 2.2	≥ 5 in geometric series	≥ 5 in geometric series	≥ 5 in geometric series	≥ 5 in geometric series	≥ 5 in geometric series	≥ 5 in geometric series	5 in geometric series of 2
Vehicle concentration	< 100 mg/L or 0.1 mL/L	< 100 mg/L or 0.1 mL/L	< 100 mg/L or 0.1 mL/L	< 100 mg/L or 0.1 mL/L	< 100 uL/L	< 0.1 mL/L	< 0.1 mL/L	< 0.1 mL/L	< 0.1 mL/L	< 0.1 mL/L	< 0.1 mL/L	NA
Endpoint(s)	96 h LC50	48 h Imm.	EC50 growth	EC50 Imm.	96 h LC50	96 h IC50 shell growth reduction	96 h LC50	96 h LC50	48 h EC50 mortality or abnormality	96 h LC50	96 h IC50 & NOEC/IC05 yield, growth rate, algal density	96 h LC50 (+ additional endpoints)

*None if water is unfiltered, feeding supplement needed if water is filtered

^Complete darkness acceptable for compounds prone to photodegradation

^&^30-minute transition recommended

^@^10 organisms preferred with 2 replicates

Building on previous work evaluating the toxicity of the UV filter benzophenone-3 (BP-3 or oxybenzone) in this species [23], the goal of this study is to investigate the utility of *G. fascicularis* as a standard test organism using three independent definitive 96 h acute copper exposures. This study will demonstrate the reproducibility of response in this species as well as provide initial information about its sensitivity in comparison to other hard coral species and utility of non-traditional additional coral biological endpoints for use in coral risk assessment.

## 2 Materials and methods

### 2.1 Test species and coral culture conditions

*G. fascicularis* were obtained from St. Mary’s College of Maryland (SMCM) from a culture system started with at least 8 individual (genetically distinct, although genetic characterization of specific lineages was not performed in this study) colonies from various sources and maintained at this location since 2002. Artificial seawater (ASW; Instant Ocean® Sea Salt Mix [Blacksburg, VA] in deionized [DI] water) was prepared at 35‰ and allowed to completely dissolve before adding it to the coral culture tanks (see Text S1, Table S1 and also Conway et al. [23] for full culture conditions and Table S2 for culture water quality parameters in all three toxicity test experiments in this study). This conditioned culture ASW was used as dilution water for all exposures. Parent colonies for each experiment were used to provide polyps for toxicological testing. Individual polyps were fragmented from the parent colony and polyps indiscriminately attached to a plain ceramic poker chip (39 mm diameter x 2.5 mm height). Polyps were allowed to recover for 3–4 weeks after fragmentation at the coral culture facility at SMCM before moving them to the Chesapeake Biological Laboratory (CBL). At CBL the polyps were acclimated to test conditions in the treatment vessels for 24 h. Coral polyps were monitored for any signs of stress during their recovery and acclimation periods. All used were visibly healthy and extended polyps. Corals were not fed during the acclimation or exposure period at CBL and subsets of corals outside of the specific tests were assessed and sacrificed on Day 0 for comparison to experimental coral biological endpoints.

### 2.2 Chemicals

Copper (II) chloride (CuCl_2_, CAS# 7447–39–4, 97%) for exposure solutions and analytical standards was obtained from Sigma-Aldrich, St. Louis, MO. For copper chemical analyses (see Section 2.5) seawater was preserved with nitric acid (Baker Instra-Analyzed, 69–70%) and digested with nitric acid and hydrogen peroxide (Baker Analyzed, 30%) as appropriate. To prepare the copper exposure solutions, a stock solution of CuCl_2_ dissolved in control water at the high concentration (1.0 mg/L copper) was prepared daily and diluted to make all other exposure concentrations. To prepare definitive test solutions, culture water for each concentration was prepared as a single solution and then split into the individual exposure vessels.

### 2.3 Experimental design

Test solution exposure was carried out in individual 2.0 L glass beakers with aeration and water circulation maintained using a bubble lift as described in Conway et al. [23] as adequate water flow is essential for the health of *G. fascicularis* [52]. To prevent evaporation and maintain salinity levels, beakers were loosely covered with plastic wrap. Glass beakers were randomly assigned for each test in a new experimental placement matrix. Ceramic poker chips with coral polyps were allocated to beakers randomly. Each concentration was readily identified using colored tape. Beakers were positioned in a water bath set to maintain a coral exposure temperature of 26 ± 1 °C which was monitored continually. EcoTech Marine Radion XR30W lights provided full-spectrum illumination (405–660 nm) for all exposures and were programmed using EcoSmart Live to follow the “Shallow Reef (Natural)” 12:12 cycle. Photosynthetically active radiation (PAR, µmol m^−2^ s^−1^) was measured daily at random locations within the experimental tank area to confirm spectral quantity. Water quality was assessed daily using pooled replicates of new and aged exposure solutions for each concentration using a YSI Professional Plus Multi-Parameter Meter (see Text S1 for details). Daily water quality and PAR are summarized in Table S2.

Before water changes each day, images of each ceramic poker chip and readings of polyps with a Junior PAM (Pulse-Amplitude-Modulation) fluorometer (Heinz-Walz, Effeltrich, Germany) were taken for analyses as described in Section 2.6. Seawater samples of pooled replicates from new and aged exposure solutions were taken daily for analytical verification of copper concentrations, algal pigment analysis and additional water quality analyses (nitrates, nitrites, ammonia, phosphates, and alkalinity; Table S3) as described in Text S1.

On day 0 additional coral polyps that were not part of the toxicity tests were photographed, measured with the Junior PAM, and immediately frozen at −80 °C for tissue analyses. Similarly, after observations were complete, all corals at the end of the 96 h definitive exposures were immediately frozen at −80 °C for tissue analysis, with these measurements detailed in Text S2.

### 2.4 Toxicity test exposure details

After preliminary range finding tests (see Text S3), copper was used for three definitive acute tests to confirm reproducibility of the toxicity tests in this coral species. Each definitive test was a 96 h, static renewal with a 24 h (daily) renewal interval including a negative control and 5 exposure concentrations (63, 125, 250, 500, and 1000 µg/L Cu) with 4 individual replicate beakers each (using 12 coral polyps per replicate, i.e. n = 3 ceramic poker chips, each containing n = 4 polyps). This test setup was based on EPA standard acute toxicity tests for invertebrate species [50]. The three definitive toxicity tests were carried out in January, February and November 2020.

### 2.5 Chemical analysis

To derive accurate toxicological thresholds it is essential that appropriate analytical verification of the toxicity test solutions is conducted [16], [17]. Analysis of dissolved and particulate copper concentrations were based on EPA 6020 guideline [53]. Seawater samples were collected in acid-washed polyethylene bottles and dissolved copper fractions (filtrate) and particulate copper fractions (material retained on the filter) were analyzed separately. Two types of exposure solutions were analyzed daily for each time point; the newly made solutions were sampled before they were added to the exposure beakers (duplicate samples were taken except for some time points in Test 3), aged solutions were pooled from all exposure chambers after the 24 h exposure for each concentration. Assessment of the particulate fraction in all of the concentrations was assessed in Test 2 to assess the potential loss of copper in the dissolved phase. To prepare the two fractions seawater was filtered through 0.45 µm cellulose acetate filters which were placed at −20 °C until digestion. The filtered samples were preserved with 0.5% nitric acid and refrigerated until analysis of dissolved copper concentrations. For dissolved analysis, samples were diluted 1 in 100 to lower the salt level and analyzed directly using an Agilent 7500 ICP-MS with helium in the collision cell to reduce interference. While internal standards were used (Germanium 72) to correct for drift, a subset of samples were spiked with copper to assess ongoing performance as suppression from complex matrices, such as saltwater, can occur. Copper 65 at. mass was used for all quantitation’s. The limit of detection (LOD) and limit of quantification (LOQ) for the dissolved copper fraction were 0.03 µg/L and 0.4 µg/L respectively. All analytical blanks for the dissolved water samples were <LOQ.

The cellulose acetate filters for analysis of the particulate copper fraction were microwave digested in a Milestone EOTHO-EZ microwave. Filters were placed in a 20 mL quartz reaction vessel with 5 mL concentrated nitric acid for 2 h, after which 5 mL DI water was added to the vessel. The vessel was covered with a quartz cap and placed inside a Teflon chamber. Ultrapure water (5 mL) and 5 mL 30% H_2_O_2_ were then added to the outer Teflon chamber. The Teflon chamber was capped and pressure sealed. The vessels were heated to 180 °C over a 20-minute period and allowed to reflux for 20 min at 180 °C. Once cooled, the samples were diluted to 15 mL with DI water. A subsample (50–500 µL) of the digest was then diluted to 10 mL and copper was measured using an Agilent 7500 ICP-MS with helium in the collision cell. Copper 65 at. mass was used for all quantitation’s and the limit of detection (LOD) and limit of quantification (LOQ) for particulate copper were 0.03 µg/L and 0.7 µg/L, respectively. Analytical blanks for the filters were <LOQ.

### 2.6 Coral biological endpoints

Coral polyps were assessed daily for a number of biological parameters, with mortality being the primary measurement to provide median lethal concentrations (LC50s) calculated as described in Section 2.7. For this species mortality was visually determined by the sloughing of tissues as well as the lack of fluorescence under the full-spectrum exposure lights (see Figure S2 in [23]). The literature describes assessing mortality in corals challenging as tissue is within the oral cavity not just externally on the skeleton. Ideally placing corals in recovery conditions after test termination can be used to verify mortality (i.e. no recovery) but as we measured pigments in the coral tissues we were unable to conduct this additional verification given the destructive nature of these sampling needs. In our range-finding and prior studies we did not see recovery after we had defined mortality in this species [23]. If mortality is overestimated this would provide a more conservative and protective assessment of the chemicals toxicity.

Because corals have a symbiotic relationship between the invertebrate host and algal symbiont, additional biological assessments common in other coral studies were used to investigate if they would be more appropriate and/or more sensitive to use for acute coral testing [17]. Daily images were used to determine the degree of polyp retraction or extension by visually scoring them from 0 (no polyps visible) to 4 (polyps appear relaxed and freely moving). This was compared to a quantification of polyp extension using Adobe Photoshop® following the methods detailed in Conway et al. [23]. Briefly, polyp extension/retraction was expressed as a ratio of polyp tentacle length and the diameter of the poker chip. As these two methods of characterizing polyp retraction correlated well [23], the quantification method was used as the endpoint of choice for statistical analyses to avoid observer bias.

Coral bleaching is a common endpoint often reported both for corals in the field and in the laboratory as an observation of stress and reduced health, often preceding mortality. In these experiments, bleaching was assessed daily during the exposures using a number of methods, including visual assessment, algal pigment analysis and number of *Symbiodinium* spp. released into the exposure solutions. First, it was visually qualified using a Coral Color Reference Card with subsequent quantification using an image analysis method modified from [54]. Images were analyzed using Adobe Photoshop® to measure brightness and saturation composition in an averaged 5 × 5 pixel selection in each polyp then averaged by replicate as detailed by Conway et al. [23]. The image analysis method was used in the statistical analyses for bleaching given its advantages in terms of reduced observer bias, better sensitivity and correlation with visual scoring as discussed previously in Conway et al. [23].

Second, the algal pigments phaeophytin and chlorophyll-a (chl-*a*) were quantified in coral tissue following Yost and Mitchelmore [55]. After defrosting from −80 °C, tissue was removed from the skeleton by air-brushing with 0.2 µm filtered ASW (at 35‰ using Crystal Sea® Marinemix [Marine Enterprises, Baltimore, MD]). The resulting slurry was homogenized and immediately processed for pigment analysis with 1 mL of the slurry filtered via GF/F, the filter was then placed into 4–5 mL of 90% acetone at 4 °C for 12–18 h before pigment quantitation as detailed in Conway et al. [23]. Subsamples of tissue slurry were frozen at −40 °C for later assessment of protein levels as described in Conway et al. [23]. Third, chl-*a* was measured in the aged exposure water to assess the loss of algae from corals into the surrounding waters by filtering exposure water onto GF/F and quantified for algal pigments as described in Conway et al. [23]. Pigment results were used in the equations outlined by NASL [56] to calculate uncorrected chl-*a*, phaeophytin, and chl-*a* corrected for phaeophytin. To determine the best manner to standardize tissue chl-*a*, both coral surface area and coral tissue protein content were quantified following the methods outlined in Conway et al. [23].

Finally, to further assess symbiont health, a Junior PAM was used to measure light-adapted photosynthetic efficiency ΔF/F_m_’ of each of the polyps using the PAM software (WinControl-3 v 3.29). Extended methods for all biological endpoints including surface area and protein calculations can be found in Text S4.

### 2.7 Statistical analysis

Median lethal and effective concentrations (LC50,ECx’s), lowest-observed effect concentrations (LOECs), and no-observed effect concentrations (NOECs) were determined. Although LOEC, NOEC, and ECx metrics are more typically applied to chronic studies, they are reported here, however, it should be noted that endpoints other than LC50s are generally used for chronic rather than acute toxicity test thresholds. These additional measures are included to understand how copper sublethally impacts this species and to identify coral-specific responses that may provide sensitive, reliable and reproducible endpoints, although their relationship to population-relevant endpoints will need to be established.

Statistical analyses were conducted in R (v. 3.4.2) and R Studio (v 1.2) with statistical significance set at the p < 0.05 level. Toxicological thresholds were calculated using measured dissolved copper concentrations from freshly prepared exposure solutions, aligning with standard environmental risk assessment and regulatory frameworks. For all endpoints except measures of chl-*a*, polyps were observed individually were treated as individual observations (n = 12 per replicate; n = 48 per concentration). For chl-*a* endpoints, each beaker (i.e. replicate) was treated as an individual observation (n = 4 per concentration). To test for significant treatment-related responses (i.e. NOEC and LOEC), methods outlined by OECD [57] were followed. The non-parametric Jonckheere trend test [58] was applied to monotonic data failing normality assumptions (evaluated via Shapiro-Wilks tests and visual Q-Q plots). For data that satisfied the assumption of normality, and were non-monotonic, Dunnett’s Test was employed.

NOEC and LOEC values were calculated using the package “mixtox” [59]. Concentration-response curves and LC/EC_x_ metrics were generated using the “drc” package ([60]; Figures S1-S5). All endpoints were treated as continuous with the exception of mortality which was expressed as percent survival at day 4 and therefore treated as a binary response with lower and upper limits of 0 and 1, respectively. All applicable models from the drc package were considered including two- to five-parameter log-normal, log-logistic, and Weibull models. The model was selected for the dose-response curve using the lowest Akaike information criterion (AIC) score in conjunction with a visual inspection of model fit.

## 3 Results and discussion

### 3.1 Exposure water chemistry

This study highlighted the critical importance of conducting analytical verification of the test exposure media over time as recommended in numerous reviews [16], [17]. Control dissolved copper concentrations were low (generally < 15 μg/L; Table S4) compared to exposure concentrations, but well above the LOQ (0.4 μg/L) showing low background levels of copper in the prepared water. Dissolved copper in newly made solutions ranged from 77 and 104% of nominal target concentrations. After 24 h, measured dissolved concentrations in aged exposure solutions decreased by 4–38% relative to newly made solutions (Table 2; Tables S4 to S6). Losses of dissolved copper are hypothesized to result from one or a number of potential reasons, including the fractionation of copper to the particulate fraction. As shown in Tables S7 and S8 (the fractionation of copper in newly made solutions and aged solutions between the dissolved and particulate fractions). In Test 2, exposure solutions were assessed for both the dissolved fraction (filtrate) and the particulate fraction (copper retained on the filter; see Tables S9 and S10). In newly made solutions across all concentrations the particulate fraction was < 2% total copper measured, whereas in the aged solutions the particulate fraction was higher, ~5–10% of the total copper measured concentration. Taking into account particulate fractions resulted in concentrations closer to nominal for new solutions (85–106%) and aged solutions (67–97%). While this partitioning accounts for some losses over 24 h, other potential loss processes are occurring and could include; adsorption to the exposure chambers or coral external surfaces, and/or biological uptake by the corals. Bioaccumulation was not quantified given the complexities in providing accurate assessment of internal uptake into coral tissues versus adsorption/binding onto coral external surfaces. Averaging new and aged exposure solutions, effective 24 h dissolved exposure concentrations ranged from 67% to 98% of nominal target values.

**Table 2:** Average daily concentrations of dissolved copper from the three toxicity tests for all four timepoints during exposures (± SD, n = 4). Newly made dissolved exposure concentrations ranged from 79% to 104% and aged dissolved exposure concentrations ranged from 51% to 92% of nominal target exposures, with the average taking into account new and the aged solutions ranging from 67% to 98% of nominal target exposures. NA = not applicable. All daily concentration averages and standard deviations are presented in Tables S4 and S5.

**Test**	**Nominal Concentration (µg/L)**	**Average New (µg/L)**	**Average % of nominal** **In New**	**Average Aged (µg/L)**	**Average % of nominal** **In Aged**	**% 24 h reduction**	**Overall New and Aged Average (µg/L)**	**Average % of nominal**
**Test 1**	**Control**	6.6 ± 0.9	NA	4.0 ± 2.9	NA	NA	5.3 ± 2.4	NA
	**63**	52.7 ± 3.2	84%	45.5 ± 8.1	72%	14%	49.1 ± 6.9	78%
	**125**	105.9 ± 5.1	85%	87.5 ± 7.7	70%	17%	96.7 ± 11.5	77%
	**250**	198.0 ± 20.5	79%	159.8 ± 15.0	64%	19%	178.9 ± 26.4	72%
	**500**	387.3 ± 34.2	77%	322.6 ± 28.7	65%	17%	355.0 ± 45.3	71%
	**1000**	768.6 ± 72.9	77%	661.7 ± 67.6	66%	14%	715.2 ± 86.6	72%
**Test 2**	**Control**	13.5 ± 2.8	NA	8.5 ± 4.2	NA	NA	11.5 ± 3.9	NA
	**63**	65.3 ± 2.3	104%	57.9 ± 6.6	92%	11%	61.6 ± 6.1	98%
	**125**	123.5 ± 9.9	99%	103.9 ± 21.6	83%	16%	113.7 ± 18.7	91%
	**250**	228.7 ± 25.2	91%	174.3 ± 25.5	70%	24%	201.5 ± 37.4	81%
	**500**	473.6 ± 67.2	95%	331.4 ± 41.5	66%	30%	402.5 ± 91.9	80%
	**1000**	838.7 ± 91.8	84%	617.2 ± 41.2	62%	26%	728.0 ± 134.6	74%
**Test 3**	**Control**	9.1 ± 5.3	NA	6.5 ± 0.9	NA	NA	7.8 ± 3.8	NA
	**63**	53.7 ± 12.8	86%	51.6 ± 4.3	82%	4%	52.6 ± 8.9	84%
	**125**	117.9 ± 17.0	94%	110.3 ± 30.3	88%	6%	114.1 ± 23.1	91%
	**250**	213.0 ± 44.1	85%	190.9 ± 39.6	76%	10%	201.9 ± 40.6	81%
	**500**	418.7 ± 95.0	84%	372.9 ± 93.2	75%	11%	395.8 ± 90.5	79%
	**1000**	828.5 ± 61.8	83%	514.1 ± 57.1	51%	38%	671.3 ± 176.9	67%

### 3.2 Mortality

All three acute definitive copper exposures produced consistent results across all biological endpoints, demonstrating the toxicological reproducibility of *Galaxea fascicularis* as a candidate for a standard toxicity test organism (Fig. 1; Figures S1-S5). The LC50s in each test (95% confidence interval) are as follows: Test 1 = 441 (321−561) µg/L; Test 2 = 547 (365−730) µg/L; Test 3 = 415 (387−445) µg/L using measured dissolved copper concentrations (Table 3, Table S11 presents the results using nominal concentrations). The LC50 averaged 468 ± 70 µg/L dissolved copper which aligns with the LC50 of 511 µg/L obtained when copper was used as a positive control during prior testing of benzophenone-3 [23] and falls within acceptability criteria for reference toxicants (i.e. +/- 2 standard deviations from the mean; [61]), which for our tests the acceptable range would be 324–548 µg/L.

**Fig. 1:**
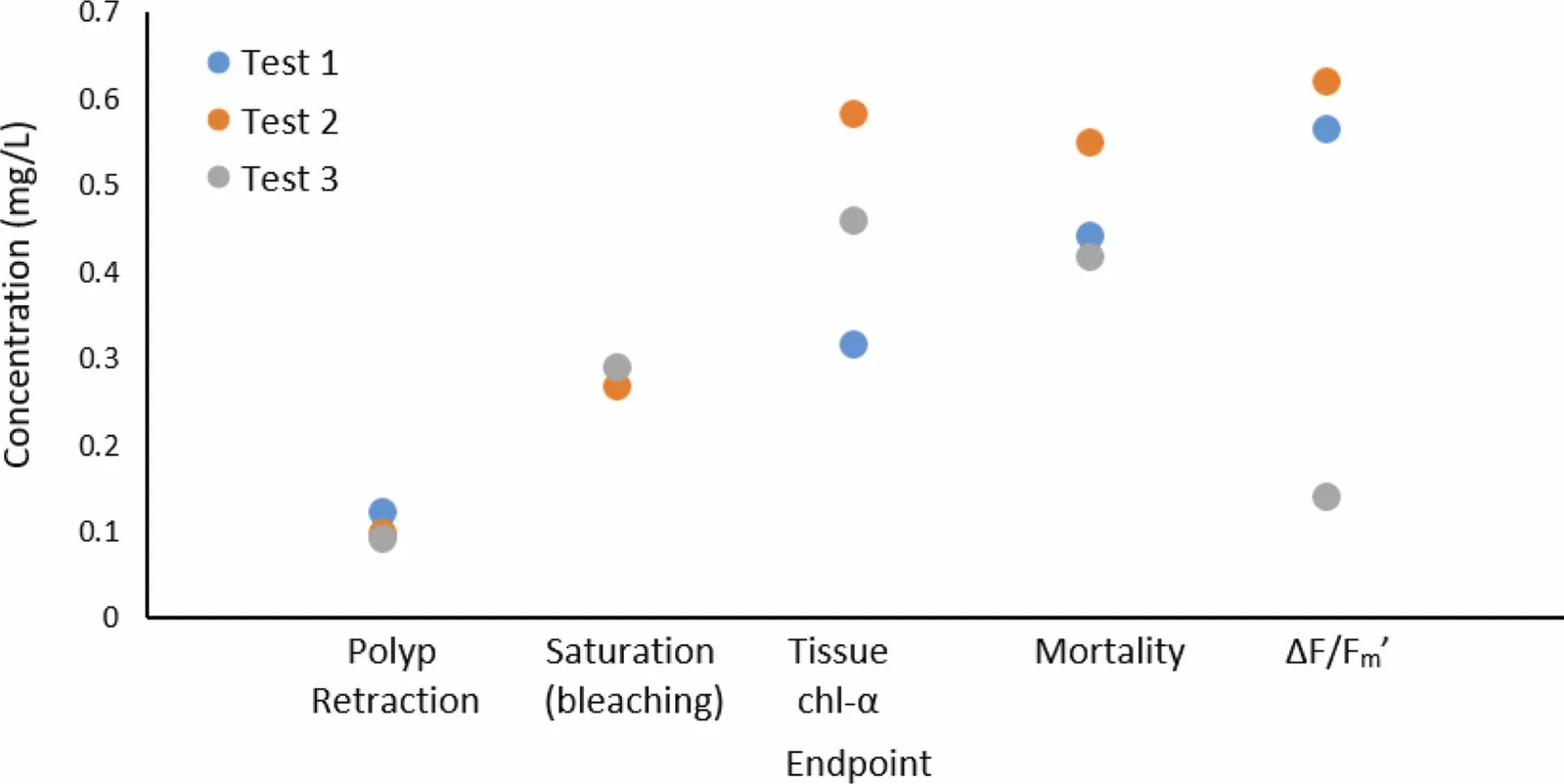
Calculated median effective/lethal concentrations (EC/LC50s) for endpoints of triplicate copper tests with *G. fascicularis*. Replication between all three tests was generally good with the exception of ΔF/F_m_’ in test 3 and some variation with tissue chl-a. Polyp retraction was seen to be the most sensitive endpoint.

**Table 3:** Summary of endpoints for repeated copper acute tests to *Galaxea fascicularis* using measured dissolved copper concentrations (*µg/*L*)*. The lowest-observed effect concentration (LOEC) is the first concentration that was significantly different (p < 0.05) from the controls while the no-observed effect concentration (NOEC) is the highest concentration not showing a significant difference from the controls. A NOEC of NA (not applicable) designates a significant difference of all testing concentrations from the controls. LCx: Lethal concentration causing X% mortality (95% confidence interval); ECx: Concentration causing and X% decline in a given measurement (95% confidence interval); DRC: Dose-response curve; J: Jonckheere Test; D: Dunnett's test; S: Steel’s Many-to-One Rank Test; LL = log-linear, LN = log-normal.

	**Endpoint**	**Test**	**P-value**	**NOEC**	**LOEC**	**DRC Model**	**LC/EC10**	**LC/EC20**	**LC/EC50**
**Test 1**	Mortality	J	< 0.001	179	355	LL.2	369 (334−404)	394 (379−409)	441 (331−553)
	ΔF/F_m_’	S	< 0.05	355	715	LN.3	378 (316−439)	433 (375−491)	563 (508−618)
	Saturation	J	< 0.001	179	355	NA	NA	NA	NA
	Polyp Retraction	J	< 0.001	NA	49	LL.5	30 (25−35)	52 (46−59)	121 (114−129)
	Tissue Chl-*a*	D	< 0.05	179	355	LL.4	140 (67−214)	189 (111−267)	314 (170−459)
**Test 2**	Mortality	J	< 0.001	229	474	LL.2	472 (455−489)	499 (438−560)	547 (365−730)
	ΔF/F_m_’	S	< 0.05	474	839	LN.3	470 (449−491)	517 (497−537)	618 (597−639)
	Saturation	J	< 0.001	65	124	LL.4	124 (87−161)	164 (132−197)	265 (226−304)
	Polyp Retraction	J	< 0.001	NA	65	LL.3	29 (24−33)	52 (45−58)	125 (117−133)
	Tissue Chl-*a*	D	< 0.05	229	474	LL.3	253 (165−341)	344 (257−430)	580 (500−659)
**Test 3**	Mortality	J	< 0.001	213	419	LL.3	334 (-29−697)	362 (108−616)	415 (395−436)
	ΔF/F_m_’	J	< 0.001	NA	54	LL.4	46 (5.5–87)	75 (28−123)	172 (64−281)
	Saturation	S	< 0.001	54	118	LL.4	85 (55−115)	133 (101−166)	289 (204−374)
	Polyp Retraction	J	< 0.001	NA	54	LL.5	11 (5.9–17)	26 (17−34)	89 (74−104)
	Tissue Chl-*a*	S	< 0.05	118	213	LL.3	33 (-4−70)	87 (19−155)	457 (260−654)

While acute copper toxicity has been investigated in other coral species, direct comparisons remain challenging due to variable methodologies and exposure durations (Table S12). Furthermore, most studies evaluated coral larvae mortality [31], [33], [38], [44], [62] with only three studies examining mortality in adult corals [33], [47], [63]. Although early life stages (i.e. coral gametes and larvae) are often more sensitive to chemical stressors, they are also more difficult to obtain and culture in a laboratory setting. In contrast, vegetative budding polyps of *G. fascicularis*, provide a continuous, year-round supply suitable for standardized testing.

Studies with coral larvae present lower 96 h LC50s for copper, ranging from 57 [31] to 107 µg/L [62] mg/L Cu (Table S7), although Hédouin et al. [33] reported a higher larval LC50 for *P. damicornis* of up to 0.198 mg/L. In adult corals studies, our observed LC50 (0.468 mg/L; Table 3) is approximately 13-fold higher (less sensitive) than the 96 h LC50 of 0.032 mg/L reported by Sabdono [47] in the same species. Limited methodological detail and lack of analytical verification in Sabdono [47] precludes determining the exact sources of this divergence. Other adult coral LC50s for copper presented in Hédouin et al. [33] for *P. damicornis* (175–251 µg/L) and Brefeld et al. [63]for *M. digitata* (161 µg/L) are more similar to our results and the authors of the latter study also noted this discrepancy with the Sabdono [47] study.

When comparing our data to current standard testing organisms relevant to corals (i.e. mysid shrimp and algae), 96 h LC50 values range from 6.97 to 284 µg/L for *Americamysis bahia* [64], [65], [66], [67]. There is only a single 96-hour algal study which is in the freshwater algal species *Chlamydomonas reinhardtii* providing an LC50 of 808 µg/L [68]. The mysid results are similar to the coral results presented elsewhere, but, again, are slightly lower than the LC50s calculated in this study. However, the microalgal results are higher than what is presented here and elsewhere for other coral species. Thus, *G. fascicularis* exhibits sensitivity intermediate between crustaceans and microalgae.

Evaluating additional genetic lineages of *G. fascicularis* and exposure to other chemical contaminants is necessary to determine broader comparative sensitivity with other hard coral species. In a previous assessment of benzophenone-3 (BP-3 or oxybenzone) using adult *G. fascicularis* an average LC50 of 6530 + /- 470 µg/L derived from three independent tests was established [23].This is higher than reported in a single coral larval test which reported a 24 h LC50 of 139 µg/L [69]. Miller et al. [19] demonstrated two widely different 48 h LC50’s in *Acropora* spp. larvae, with *A. digitifera* significantly more sensitive (0.75 µg/L) than *A. millepora* at 1042.31 µg/L. He et al. [70] was unable to calculate an LC50 for the 2 species of adult coral fragments exposed to 1 mg/L BP-3 as no or limited mortality occurred following 7 days exposure. These results highlight the challenges of comparisons between species given the limited number of studies available, different coral life stages, and particularly the differing methodological designs, quality control measures and lack of analytical chemical assessments, issues previously noted with many coral toxicity studies [8], [16], [17]. Recently Raimondo et al. [10] highlighted this issue concluding that only 4% of coral UV filter toxicity data were quantitatively acceptable for inclusion in an environmental risk assessment. Therefore, it is essential and the goal of this study to provide clear guidelines for a standard toxicity test species, including test design, minimal data reporting and appropriate analytical verification of the actual exposure solutions throughout the duration of the study. Furthermore, even if this species is hardier than others and consistent across chemical contaminant classes adjustment factors could be applied to toxicity testing results to account for more sensitive coral species. It also provides an additional species of a different taxa to include in species sensitivity distributions.

### 3.3 Additional coral biological endpoints

Standard acute toxicity tests may also have additional observations recorded outside of death or immobilization. For example, the EPA OCSPP 850.1075: Freshwater and Saltwater Fish Acute Toxicity Test requires that “any abnormal behavior or appearance, and the number of individuals exhibiting these characteristics, should be counted and recorded at the same time as observations of mortality” [71]. For corals, an obvious behavioral change is polyp retraction. Polyp retraction has often been reported as a behavioral sign of stress with many triggers (e.g. sedimentation stress: [72]; light stress: [73]) including copper exposure [33] and [63]. Long-term polyp retraction may reduce feeding for a heterotrophic species like *G. fascicularis* which would in turn decrease nutrient intake [74]. Furthermore, this behavior is energetically expensive and may shade symbionts, decreasing their carbon fixation and leading to decreased growth if sustained over longer periods of time. These long-term effects could be exacerbated by negative impacts from the stressor to the symbionts directly. Although this endpoint is commonly used as an early stress biomarker, results are variable between species and chemical contaminants and its population relevance requires further investigation for use as an endpoint that can be used in regulatory toxicity tests [17]. In our exposures, polyp retraction exhibited a clear dose-dependent increase across all copper exposure concentrations (Figure S4). Given its sensitivity, dose-dependent response, and potential energetic impacts, polyp retraction warrants consideration as an additional standard sublethal endpoint in chronic toxicity testing or simply as a supplementary behavioral observation in acute testing.

Bleaching and measures of symbiont health are widely reported endpoints in coral toxicity studies [17]. An increase in visible bleaching and a decline in photosynthetic efficiency of the symbionts (i.e. ΔF/F_m_’ decline) did not occur in polyps that were not showing signs of severe stress and imminent mortality. Therefore, copper seems to more quickly and severely affect the host rather than the algal symbiont which mirrors the aforementioned toxicity thresholds from standard algal testing and aligns with the findings presented in Jones et al. (2004) and Cheng et al. [75]. The EC50 of ΔF/F_m_’ was generally similar to the LC50 and the LOEC/NOEC was identical to those for mortality in the first two tests but gave quite different results in the third possibly due to a slight decrease in laboratory light intensity. Furthermore, Bielmyer et al. [27] and Banc-Prandi et al. [26] studied the effects of copper to the effective quantum yield (equivalent to light-adapted photosynthetic efficiency) and showed significant impacts *Acropora cervicornis* zooxanthellae at 20 µg/,to *P. damicornis* at 4 µg/L [27], and to *Stylophora pistillata* at 1 µg/L [26] which shows some moderate variation between species.

Multiple studies have examined algal endpoints including chl-*a* ([76] [24 h NOEC: 11 µg/L], [26] [3 day LOEC: 1 µg/L], and [77] [14 h LOEC: 30 µg/L]), algal cell density ([78] [48 h LOEC 5 µg/L]), and zooxanthellae density ([35] [48 h LOEC: 20 µg/L). Our studies showed tissue chl-*a* concentrations (per mm^2^) significantly decreasing at 213 µg/L and higher and saturation decreasing at 118 µg/L and higher (Table 3) suggesting some level of bleaching, or loss of algal symbionts, at these dissolved copper concentrations. Further suggestive of the loss of algae from corals is the increase in chl-*a* levels in the aged exposure water (i.e. 24 h old) in all tests. Chl-*a* levels showed dose-dependent increases for the first two days then a subsequent decline to concentrations similar to the control treatment (Figure S6). Therefore, *G. fascicularis* may be more resistant to copper than other coral species studied to date. Tissue chl-*a* and image analysis of saturation percentages were both highly replicable endpoints in the three experiments conducted in this study with tissue chl-*a* appearing more variable and slightly less sensitive than saturation measurements in two of three tests. However, in Test 1, the saturation EC50 was calculated as 4010 µg/L which is higher than any other endpoint calculation likely because little variation in saturation was seen in this test. Even considering this, saturation analysis can be completed relatively quickly and has the capability of being highly repeatable. Because of the ease of analysis, saturation percentage determination may be better suited for routine toxicity testing. However, tissue chl-*a* analysis could be added in the event of a compound with a suspected bleaching mode of action or compounds in which preliminary testing showed significant impacts to saturation measures to confirm bleaching.

The results in this study lead to important questions of interspecies sensitivity and intraspecies variability when compared to literature values. From these results, *G. fascicularis* seems to potentially be less sensitive than other hard coral species, at least following exposure to copper in this study. Hédouin et al. [33] suggested difference in copper toxicity may be influenced by long-term historical contaminant exposure. Copper acclimation has been achieved in both algae (*Pseudokirchneriella subcapitata*: [79], [80]) and invertebrates (*Daphnia magna*: [80], [81], [82], [83]; *Tigriopus japonicas*: [84]) up to 1.7 times the original 48h-LC50 over 3 generations of acclimation. There are very few other contaminant studies with this species thereby limiting broader species comparisons with other corals and invertebrate species. Previously we conducted acute toxicity tests with benzophenone-3 in *G. fascicularis* [23] but given limited studies with other corals we were unable to assess its relative sensitivity as discussed in Section 3.2.

To investigate the species sensitivity of *G. fascicularis* further, a species sensitivity distribution (SSD) was attempted using the information from the EPA ECOTOX database ([85]; Figure S7). Unfortunately, the only endpoint in this database for *G. fascicularis* is from Sabdono [47]. Although these compounds have been tested on a variety of other species, there are no other calculated adult LC50s with which to compare. In the case of the additional endpoints, it is impossible to compare more than a couple of species due to differences in test duration, life stage, and endpoints measured. Recently, data from Turner et al. [51] and Renegar and Turner [86] demonstrated exposure of five species of corals to three different hydrocarbons in an effort to determine differences in coral species sensitivity. Unfortunately, *G. fascicularis* was not part of this study, but this does provide a framework which is necessary moving forward to determine relative sensitivity of different species and relative toxicity of different compounds without an accepted, standardized coral testing approach.

In order to approximate relative sensitivity, a combined ranking of several studies which compared at least three species was used to build upon “hardiness” rankings from [87]; Table 4). This includes the aforementioned studies on hydrocarbons [51], [86]), a study on the effects of temperature and pCO_2_ [88], a study on the combined impacts of UV light and temperature [89], and a final study on temperature sensitivity [90]. As Sprung [87] ranked hardiness on a scale of 1–10, the least sensitive genus in each study was given a rank of 10 (i.e. hardiest) and those ranked as more sensitive were given subsequently lower numbers. This assumes the genus which was least sensitive to the stressor is actually the least sensitive of all genera; however, this was necessary in order to provide average rankings and is only used as a tool to help determine where *Galaxea spp.* sensitivity lies. Only genera with at least three rankings (other than [87]) were considered, except in the case of *Galaxea spp.* where only one other study ranked the genus of interest. With this ranking system, *Galaxea spp.* achieves an average ranking in the middle of the pack, being the 5th most sensitive genus out of 10 although results for this study would suggest it is far less sensitive than others. However, this ranking is to be taken lightly as there is little data on this genus and all studies used in this ensemble have variable methodology. This again highlights the need for standardized coral testing following the established and openly available procedures and guidelines that OECD and the US EPA provides. Using this framework, *G. fascicularis* can then be assessed next to a number of other coral species to determine what assessment factors would need to be put in place in order to take into account this species sensitivity, or lack thereof, in comparison to other reef-building corals.

**Table 4:** Sensitivity ranking of 10 hard coral genera based on multiple stressors.

	[87]	[51]**and**[86]			[88]			[89]	[90]		
**Genus**	**Overall**	**Toluene**	**Methyl-** **napthalene**	**Phenan-threne**	**Temp.**	**pCO**_**2**_	**Temp. + pCO**_**2**_	**UV light + Temp.**	**Temp.**	**Sum**	**Avg. Ranking**
*Siderastrea*	6	7	9	10						32	8
*Acropora*	4.7*	8	8	10				8	10	48.7	8.12
*Porites*	7	6	10	8	8	8	8		10	65	8.13
*Pocillopora*	7				8	9	9		8	41	8.2
*Galaxea*	7								10	17	8.5
*Solenastrea*	7	9	10							26	8.67
*Stephanocoenia*	9	10	7	9						35	8.75
*Montipora*	6				9	10	10	10	8	53	8.83
*Fungia*	8				10	10	10			38	9.5
*Leptastrea*	9				10	10	10			39	9.75

* Sprung [87] gave 3 hardiness scores that were species-dependent. This is the average value of those 3 scores (3, 4, 7).

## 4 Conclusions

Based on exposure to copper *Galaxea fascicularis* offers an accessible, reliable and highly reproducible model organism for scleractinian toxicity testing. As a global shallow-water species, *G. fascicularis* is an ecologically relevant species for toxicity testing for coastal chemical contaminants *G. fascicularis* grows well in laboratory culture conditions and is easily fragmented allowing laboratories without direct access to natural seawater to test on a coral species. Acute copper toxicity thresholds established here bracketed traditional invertebrate and algal test species. This species maybe less sensitive to contaminants compared to other coral species, although this is difficult to assess given the sparse body of literature available and very different methodologies employed between studies. Additional testing using alternate strains of this species and exposure to other commonly tested contaminants in other coral species will be needed before its utility as a standard test organism can be established. This standard toxicity testing framework modified from OECD/US EPA invertebrate test guidelines provided robust results and should be used in further testing to make coral toxicity endpoints comparable and sensitivity rankings possible. The additional sublethal biological endpoints should be further evaluated as sensitive and population relevant endpoints besides mortality. Furthermore, a standard test using a hard coral like *G. fascicularis* should be considered by EPA and OECD due to the ecological importance of reef-building corals.

## CRediT authorship contribution statement

**Annaleise J. Conway:** Writing – original draft, Validation, Methodology, Investigation, Formal analysis, Data curation, Conceptualization. **Andrew Heyes:** Writing – review & editing, Validation, Methodology, Investigation, Formal analysis. **Carys L. Mitchelmore:** Writing – review & editing, Writing – original draft, Visualization, Validation, Supervision, Resources, Project administration, Methodology, Investigation, Funding acquisition, Formal analysis, Data curation, Conceptualization.

## Funding

Funding for this research was provided by 10.13039/100004331Johnson and Johnson Consumer Inc. as a standard academic research grant awarded to UMCES [Sponsor Grant # 1301058] in full compliance with University regulations regarding academic freedom and intellectual property. The contents of this paper are solely the responsibility of the authors. This paper is contribution number 6527 of the University of Maryland Center for Environmental Science (Ref. No. [UMCES] CBL 2026-056).

## Declaration of Competing Interest

The authors declare that they have no known competing financial interests or personal relationships that could have appeared to influence the work reported in this paper.

## Data Availability

Data will be made available on request.
