# Supplementary material for: Acute toxicity of copper to Galaxea fascicularis and its utility as a candidate standard coral toxicity test species
Source: Toxicol Rep. 2026 Oct 28;17:102363. doi: 10.1016/j.toxrep.2026.102363 (PMC13756509; doi:10.1016/j.toxrep.2026.102363)
Supplement: Supplementary file 1 [file mmc1.pdf]

Acute toxicity of copper to *Galaxea fascicularis* and its utility as a candidate standard coral toxicity test species

Annaleise J. Conway[1], Andrew Heyes, Carys L. Mitchelmore*

University of Maryland Center for Environmental Science, Chesapeake Biological Laboratory, 146 Williams Street, Solomons, MD 20688, USA.

*** Corresponding Author:**

Carys L. Mitchelmore,

Email: mitchelmore@umces.edu

Address: University of Maryland Center for Environmental Science, Chesapeake Biological Laboratory, 146 Williams Street, Solomons, MD 20688, USA.

Present address for Annaleise J. Conway:

[1]Integral Consulting Inc., 200 Harry S. Truman Parkway Suite 330, Annapolis, MD 21401, USA

Content in Supplemental Information

Page Number

Text

Text S1. Coral Culture Conditions…………………………………………………….….4

Text S2. Test Setup Additional Details…………………………………………….……..4

Text S3. Preliminary Toxicity Testing (range-finders)…………………………….……..5

Text S4. Coral Biological Endpoints Additional Details……………………………………5-6

Tables

Table S1. Water quality parameters for coral culture during time of testing……….……..7

Table S2. Summary of water quality parameters and light quantity measured daily………..8

Table S3. Summary of additional water quality parameters measured during testing...…..…9

Table S4. Average daily concentrations of new dissolved copper exposure solutions from the three toxicity tests for all four timepoints during exposures……………10

Table S5. Daily concentrations of aged copper exposure solutions………………………....11

Table S6. Differences in Dissolved copper concentration and percentage reduction in aged copper exposure solutions compared to the corresponding new solutions......................................12

Table S7. Daily concentration of particulate copper in new exposure solutions in Test 2…..13

Table S8. Daily concentration of particulate copper in aged exposure solutions in Test 2….14

Table S9. Average daily particulate, dissolved and total copper concentrations in newly made exposure solutions from all four timepoints in Test 2………………………15

Table S10. Average daily particulate, dissolved and total copper concentrations in aged Exposure solutions from all four timepoints in Test 2…………………………...16

Table S11. Summary of Endpoints for repeated copper acute tests to *Galaxea fascicularis* using nominal dissolved copper concentrations (µg/L)…………………………17-18

Table S12. Calculated median lethal concentrations (LC50s) for coral species of all life stages……………………………………………………………………19-20

Figures

Figure S1. Dose response curves for survival (mortality)…………………………………..21

Figure S2. Dose response curves for tissue chl-*a*……………………………………….....22

Figure S3. Dose response curves for photosynthetic efficiency……………………………23

Figure S4. Dose response curves for polyp retraction………………………………………24

Figure S5. Dose response curves for saturation……………………………………………..25

Figure S6. Chlorophyll-*a* concentrations in aged exposure solutions……………………….26

Figure S7. Species sensitivity distribution (SSD) for copper modeled using a normal and logistic model…………………...……………………………….27-28

References

Reference list…………………………………………………………………………..29

Supplementary Text

Text S1. Coral Culture Conditions

Details of the coral artificial seawater makeup, coral culture conditions, source and genetic variability of parent fragments of *Galaxea fascicularis* from St. Mary's College of Maryland (SMCM) are provided in Text S1 in Conway et al. (2021). This culture water was used as control water and to make experiment exposure dosing solutions. Appropriate facilities for coral toxicity testing and chemical analysis were not available at SMCM so testing took place at Chesapeake Biological Laboratory. Travel between the two locations is ~30 minutes and corals are transported in a small cooler to maintain temperature and moisture levels.

Text S2. Test Setup Additional Details

The 4-star coral polyp attachment to ceramic poker chips was chosen to allow for easy observations on individual polyps. A number of orientations were attempted, however attaching the polyps standing up was physically challenging and keeping polyps attached to each other made differentiation of polyps and standardization of polyp numbers on each chip difficult.

Exposures were carried out in 2.0 L glass beakers containing an attached glass tube on the inner wall of the beaker so that a glass pipette attached to an airline could generate bubbles to drive water circulation and maintain dissolved oxygen levels in the exposure solutions (see Conway et al. 2021 for a diagram). Glass beakers for all tests were randomly placed in a water bath at a temperature of 26 °C ± 1° C which was monitored using a HOBO Pendant® MX Water Temperature Data Logger (Onset, MA). Using a YSI Professional Plus Multi-Parameter Meter (YSI, Inc. Yellow Springs, OH), water quality assessments were performed daily on pooled replicates of new (i.e. immediately after solution preparation) and aged (i.e. after 24 h of coral exposure, before renewal) solutions for each copper concentration.

Before daily water changes, images of each chip were taken using a Canon EOS 60D camera with a macro lens for the analyses described below and light adapted photosynthetic efficiency measured using a Junior Pulse-Amplitude-Modulation (PAM) fluorometer (Heinz-Walz, Effeltrich, Germany). Daily replicates of newly made up seawater for each exposure concentration

were pooled as were samples of aged seawater from each treatment prior to the new addition. Samples for analytical confirmation of new (n = 2 per concentration) and aged (n = 1) exposure concentrations were refrigerated until processing and analysis as described in the main manuscript and Conway et al. (2021). Additional daily new and aged seawater samples from each concentration were filtered through a 0.7 µm GF/F glass fiber filter. The filter and filtrate were independently frozen at -80 °C for chlorophyll-α/phaeophytin analysis and additional water quality analyses (nitrates, nitrites, ammonia, phosphates, and alkalinity), respectively.

Text S3. Preliminary Toxicity Testing (range-finders)

To determine the concentration range for definitive testing, three preliminary range-finding acute toxicity tests were carried out without replication of exposure vessels. Copper toxicity tests were conducted using $CuCl_2$ (10,000, 1,000, 100, 10, 1 µg/L Cu; test 1 and 1,000, 500, 100, 50, 10 µg/L Cu; test 2) or $CuSO_4$ (10,000, 1,000, 100, 10, 1 µg/L Cu; test 3) plus a negative control (dilution seawater only) for each test with 4 polyps in each replicate. The toxicity observed in the two copper compounds was comparable so $CuCl_2$ was employed for definitive testing because it dissolved more quickly in seawater than $CuSO_4$. Total mortality in all tests was observed in the 10ml/L and 1mg/L doses and partial mortality at 500µg/L. These results refined the concentrations used for the acute definitive tests, complying with US EPA and OECD acute toxicity test concentration spacing to be a maximum of 2 or 2.2 times (US EPA, 2016b; OECD, 2025). The concentrations used for all three definitive tests employed a 2x spacing and were (63, 125, 250, 500, and 1,000 µg/L Cu).

Text S4. Coral Biological Endpoints Additional Details

Detailed descriptions of how each biological endpoint was qualitatively or quantitatively assessed can be found in the supplementary material provided in Conway et al. (2021). Briefly, polyp retraction/extension was assessed by visual scoring or using image analysis of daily photographs. Coral bleaching was assessed in a number of ways, visually using color reference cards, via image analysis of photos, the quantitation of algal pigments chl-*a* and phaeophytin in coral tissue or in exposure water. Both protein and surface area were quantified as potential

standardization methods for pigment content. This allowed us to investigate the relationship with total protein versus surface area of the polyp and help determine if the toxicant results in overall tissue loss. Total protein content was determined using the Pierce™ BCA method (Thermo Fisher Scientific, Waltham, MA). Briefly, the tissue homogenate was defrosted, re-homogenized, and 100 µL was added to 200 µL of 0.2 µm filtered ASW (i.e. a 1:3 dilution). Methods were conducted as outlined in the BCA protocol with 25 µL of the diluted tissue sample added to triplicate wells. Surface area was calculated in a manner similar to quantitative polyp retraction. Pictures of coral skeletons after tissue removal were taken. Using Adobe Photoshop®, each polyp skeleton was measured using the "measure" tool along the central length and width. These measurements were used to calculate the surface area of a cylinder, an approximation of the surface area of the coral polyp skeleton.

For light-adapted photosynthetic efficiency determination, the last 2 inches (~5 cm) of the 50 cm plastic fiber light guide were covered in black tape to decrease additional light input from the sides. If $\Delta F/F_m$' was below 0.200, the reading was re-measured to confirm. Measurements were taken when corals were removed for the daily water change by holding the plastic fiber light guide approximately 2 mm from the surface of the polyp near its center. One measurement was taken per polyp (n = 12 polyps per replicate, 48 polyps per concentration). On Day 0 for all definitive tests, PAM was conducted on all polyps of the representative corals and also done on one polyp of each poker chip of the exposure concentrations and controls.

Supplementary Tables

Table S1. Water quality parameters for coral culture during time of testing. SD = standard deviation.

	**Phosphorus (mg/L)**	**Nitrate (mg/L)**	**Iodine (mg/L)**	**pH**	**Temperature (°C)**	**Salinity (‰)**
**Average**	0.306	0.020	0.115	7.82	25.37	36.00
**SD**	0.124	0.049	0.121	0.05	0.54	0.72

Table S2. Summary of water quality parameters and light quantity measured daily. Parameters given as a range (average) during each test. DO = dissolved oxygen.

**Test**	**Light (μmol m^{-2} s^{-1})**	**Temp. (°C)**	**Salinity (‰)**	**DO (mg/L)**	**DO (%)**	**pH**
**Test 1**	108 - 210 (155)	24.2 - 26.8 (25.2)	33.98 - 36.08 (34.95)	5.64 - 6.93 (6.10)	77.0 - 89.8 (85.9)	7.64 - 8.09 (7.89)
**Test 2**	89 - 209 (135)	24.7 - 26.1 (25.6)	33.06 - 34.33 (33.80)	5.27 - 6.15 (5.68)	78.1 - 87.0 (83.4)	7.63 - 8.08 (7.88)
**Test 3**	94 – 171 (128)	24.6 - 25.5 (25.1)	31.91 – 32.51 (32.21)	6.00 - 7.02 (6.68)	81.1 - 102.4 (95.6)	7.72 - 8.44 (8.20)

Table S3. Summary of additional water quality parameters measured during testing. Parameters given as average ± standard deviation during each test.

Test	NH_4^+ (mg/L)	NO_2^- (mg/L)	PO_4^{3-} (mg/L)	NO_3^- (mg/L)	Alkalinity (dKH)
**Test 1**	0.13 ± 0.11	0.005 ± 0.002	1.04 ± 0.53	0.014 ± 0.011	4.98 ± 0.40
**Test 2**	0.05 ± 0.07	0.005 ± 0.002	1.36 ± 0.21	0.048 ± 0.081	4.60 ± 0.39
**Test 3**	0.02 ± 0.03	0.004 ± 0.001	0.31 ± 0.13	0.030 ± 0.017	4.77 ± 0.56

Table S4. Average daily concentrations of new dissolved copper exposure solutions from the three toxicity tests for all four timepoints during exposures. Results are presented as the two pooled replicates from all exposures for each analysis with average in parenthesis, except test 3 which for Day 2-4 is one pooled sample from all exposure chambers.

Test	Nominal Concentration (µg/L)	Day 1 Concentration (µg/L)	Day 2 Concentration (µg/L)	Day 3 Concentration (µg/L)	Day 4 Concentration (µg/L)
**Test 1**	**Control**	7.8, 8.0 (7.9)	5.9, 6.9 (6.4)	6.0, 6.4 (6.2)	5.0, 6.0 (5.7)
	**63**	48.1, 50.7 (49.4)	55.2, 56.6 (55.9)	54.3, 55.4 (54.9)	51.0, 50.3 (50.6)
	**125**	98.3, 100.8 (99.6)	104.8, 110.5 (107.7)	110.0, 113.5 (111.7)	103.6, 105.4 (104.5)
	**250**	215.5, 197.5 (206.5)	204.4, 202.1 (203.2)	213.0, 215.8 (214.4)	168.5, 167.6 (168.0)
	**500**	378.5, 383.9 (381.2)	403.6, 406.8 (405.2)	421.9, 419.5 (420.7)	342.5, 342.0 (342.2)
	**1000**	723.6, 732.2 (727.9)	809.0, 813.5 (811.3)	849.5, 843.8 (846.7)	690.5, 686.8 (688.6)
**Test 2**	**Control**	7.7, 11.7 (9.7)	13.7, 12.3 (13.0)	22.4, 10.2 (16.3)	12.2, 17.4 (14.8)
	**63**	56.6, 70.3 (63.4)	58.7, 67.6 (63.1)	66.9, 681.9 (66.9)	70.5, 64.9 (67.7)
	**125**	112.8, 122.3 (117.6)	117.5, 108.6 (113.1)	139.4, 118.4 (128.9)	129.3, 139.6 (134.4)
	**250**	191.7, 323.3 (257.5)	192.1, 199.9 (196.0)	233.4, 225.7 (229.6)	246.8, 216.8 (231.8)
	**500**	658.4, 446.1 (552.2)	380.9, 397.7 (389.3)	448.8, 482.8 (465.8)	469.5, 504.3 (486.9)
	**1000**	757.8, 737.0 (747.4)	771.6, 775.6 (773.6)	889.0, 914.9 (901.9)	933.3, 930.8 (932.0)
**Test 3**	**Control**	4.9, 4.1 (4.5)	6.0	9.5	16.3
	**63**	38.4, 38.6 (38.5)	66.2	62.1	48.1
	**125**	120.9, 83.9 (102.4)	137.4	126.8	104.9
	**250**	169.3, 152.7 (161.0)	264.2	229.2	197.5
	**500**	300.1, 309.4 (304.7)	520.9	466.9	382.3
	**1000**	715.8, 1005.4 (860.6)	876.1	838.6	738.7

Table S5. Daily concentrations of aged dissolved copper exposure solutions. These are from the three toxicity tests for all four timepoints during exposures, using pooled exposure solutions from all replicate beakers after 24 hours in the beakers and before the next exposure solution change. - = no data.

Test	Nominal Concentration (µg/L)	Day 1 Concentration (µg/L)	Day 2 Concentration (µg/L)	Day 3 Concentration (µg/L)	Day 4 Concentration (µg/L)
Test 1	**Control**	0.7	7.0	5.8	2.4
	**63**	38.6	54.5	50.2	38.6
	**125**	80.1	94.2	94.2	81.6
	**250**	141.6	174.3	169.8	153.7
	**500**	287.6	348.0	344.2	310.7
	**1000**	666.3	645.6	749.3	585.8
Test 2	**Control**	-	11.4	5.5	-
	**63**	52.5	52.0	65.0	62.0
	**125**	81.2	92.7	130.3	111.4
	**250**	140.6	170.7	185.8	200.1
	**500**	281.3	317.0	349.9	377.2
	**1000**	576.4	616.6	-	658.7
Test 3	**Control**	5.3	6.7	6.4	7.5
	**63**	48.8	54.0	56.3	47.1
	**125**	68.3	118.0	114.4	140.6
	**250**	136.9	221.1	220.1	185.5
	**500**	245.0	454.7	427.0	364.7
	**1000**	598.8	492.5	473.8	491.1

Table S6. Differences in dissolved copper concentration and % reductions (in parenthesis) in aged copper exposure solutions compared to the corresponding new solution. Comparisons are over each 24 h period (positive values are reductions, negative values are higher values than the new solutions). Aged samples are taken after 24 hours of adding the new solutions and so just before the re-dosing for the next Day. - = no data.

Test	Nominal Concentration (µg/L)	Day 1 Concentration (µg/L) and % change	Day 2 Concentration (µg/L) and % change	Day 3 Concentration (µg/L) and % change	Day 4 Concentration (µg/L) and % change
Test 1	**Control**	-	-	-	-
	**63**	10.8 (21.8%)	1.4 (2.5%)	4.7 (8.6%)	12.0 (23.7%)
	**125**	19.5 (19.5%)	13.5 (12.5%)	17.6 (15.7)	22.9 (22.0%)
	**250**	64.9 (31.4%)	28.9 (14.2%)	44.6 (20.8)	14.4 (8.6%)
	**500**	93.6 (24.6%)	57.2 (14.1%)	76.5 (18.2)	31.6 (9.2%)
	**1000**	61.6 (8.5%)	165.6 (20.4%)	97.4 (11.5)	102.9 (14.9%)
Test 2	**Control**	-	-	-	-
	**63**	11.0 (17.3%)	11.1 (17.6%)	1.9 (2.9%)	5.7 (8.4%)
	**125**	36.4 (30.9%)	20.4 (18.0%)	-1.4 (-1.1%))	23.0 (17.1%)
	**250**	116.9 (45.4%)	25.3 (12.9%)	43.8 (19.1%)	31.7 (13.7%)
	**500**	271.0 (49.1%))	72.2 (18.6%)	115.9 (24.9%)	109.7 (22.5%)
	**1000**	171.1 (22.9%)	156.9 20.3%)	-	273.3 (29.3%)
Test 3	**Control**	-	-	-	-
	**63**	-10.3 (-26.7%)	12.2 (18.4%)	5.8 (9.3%)	1.0 (2.1%)
	**125**	34.1 (33.3%)	19.5 (14.2%)	12.4 (9.8%)	-35.7 (-34.0%)
	**250**	24.1 (15.0%)	43.1 (16.3%)	9.1 (4.0%)	12.1 (6.1%)
	**500**	59.7 (19.6%)	66.2 (12.7%)	39.9 (8.5%	17.6 (4.6%)
	**1000**	261.7 (30.4%)	383.6 (43.8%)	364.7 (43.5%)	247.6 (33.5%)

Table S7. Daily concentrations of particulate copper in new exposure solutions for Test 2. Results are for all four timepoints during exposures and presented as the two pooled replicates from all exposures for each analysis with average in parenthesis. - = no data.

Nominal Concentration (µg/L)	Day 1 Concentration (µg/L)	Day 2 Concentration (µg/L)	Day 3 Concentration (µg/L)	Day 4 Concentration (µg/L)
**Control**	0.05, 0.18 (0.12)	0.06, 0.39 (0.22)	-	0.37, 0.14 (0.26)
**63**	0.46, 0.55 (0.51)	1.94, 1.89 (1.91)	1.07, 0.64 (0.86)	1.69, 1.15 (1.42)
**125**	0.63, 0.71 (0.67)	3.87, 2.67 (3.27)	1.17, 0.83 (1.00)	1.28, 1.02 (1.15)
**250**	1.60, 2.44 (2.02)	1.90, 2.13 (2.01)	1.52, 1.81 (1.66)	2.62, 3.27 (2.94)
**500**	3.11, 4.98 (4.05)	3.44, 3.41 (3.43)	4.46, 2.95 (3.71)	3.72, 3.46 (3.59)
**1000**	7.95, 12.65 (10.30)	7.47, 7.49 (7.48)	10.07, 12.10 (11.09)	13.44, 13.33 (13.39)

Table S8. Daily concentrations of particulate copper in aged exposure solutions for Test 2. Results are for all four timepoints during exposures and presented as the pooled sample from all exposures. - = no data.

Nominal Concentration (µg/L)	Day 1 Concentration (µg/L)	Day 2 Concentration (µg/L)	Day 3 Concentration (µg/L)	Day 4 Concentration (µg/L)
**Control**	-	-	-	-
**63**	3.3	1.9	2.7	4.5
**125**	4.3	4.6	5.2	7.3
**250**	7.0	29.7	14.0	12.0
**500**	38.8	29.6	28.3	23.0
**1000**	94.7	57.3	59.4	57.3

Table S9. Average daily particulate, dissolved and total copper concentrations in newly made exposure solutions from all four timepoints for Test 2. Total copper concentrations (dissolved and particulate) are presented as % of nominal target concentration and the percentage of the particulate fraction making in the total copper concentration is calculated.

Concentration (µg/L)	Dissolved (µg/L)	Particulate (µg/L)	Total Copper (µg/L)	Particulate Fraction (%)	Total % of nominal
Control	13.5 ± 2.84	0.20 ± 0.07	13.6 ± 2.82	1.53 ± 0.30	NA
63	65.3 ± 2.34	1.17 ± 0.62	66.6 ± 2.38	1.76 ± 0.94	105.5 ± 3.77
125	123.5 ± 9.89	1.52 ± 1.18	125.0 ± 9.26	1.25 ± 1.05	100.0 ± 7.41
250	228.7 ± 25.2	2.16 ± 0.55	230.9 ± 25.3	0.94 ± 0.24	92.3 ± 10.10
500	473.6 ± 67.2	3.69 ± 0.26	477.3 ± 67.4	0.78 ± 0.07	95.5 ± 13.5
1000	838.7 ± 91.8	10.56 ± 2.44	849.3 ± 93.7	1.24 ± 0.20	84.9 ± 9.37

Table S10. Average daily particulate, dissolved and total copper concentrations in aged exposure solutions from all four timepoints for Test 2. Total copper concentrations (dissolved and particulate) are presented as % of nominal target concentration and the percentage of the particulate fraction making in the total copper concentration is calculated. -: no data.

Concentration (µg/L)	Dissolved (µg/L)	Particulate (µg/L)	Total Copper (µg/L)	Particulate Fraction (%)	Total % of nominal
Control	5.63 ± 5.70	-	-	-	-
63	57.9 ± 6.61	3.10 ± 1.10	61.0 ± 7.13	5.05 ± 1.54	96.8 ± 11.3
125	103.9 ± 21.6	5.35 ± 1.35	109.3 ± 22.3	4.94 ± 0.95	87.4 ± 17.8
250	174.3 ± 25.5	15.7 ± 9.80	190.0 ± 28.8	8.06 ± 4.60	76.0 ± 11.5
500	331.4 ± 41.5	29.9 ± 6.57	361.3 ± 35.2	8.47 ± 2.69	72.3 ± 7.04
1000	617.2 ± 41.3	67.2 ± 18.4	687.0 ± 25.2	10.2 ± 3.39	68.7 ± 2.5

Table S11. Summary of endpoints for repeated copper acute tests to Galaxea fascicularis using nominal dissolved copper concentrations (µg/L). The lowest-observed effect concentration (LOEC) is the first concentration that was significantly different ($p < 0.05$) from the controls while the no-observed effect concentration (NOEC) is the highest concentration not showing a significant difference from the controls. A NOEC of NA (not applicable) designates a significant difference of all testing concentrations from the controls. LCx: Lethal concentration causing X% mortality; ECx: Concentration causing and X% decline in a given measurement; DRC: Dose-response curve; J: Jonckheere Test; D: Dunnett's test; S: Steel's Many-to-One Rank Test; LL = log-logistic, LN = log-normal; W = Weibull.

	Endpoint	Test	P-value	NOEC	LOEC	DRC Model	LC/EC10 (95% CI)	LC/EC20 (95% CI)	LC/EC50 (95% CI)
Test 1	Mortality	J	<0.001	250	500	LL.2	476 (432-520)	509 (490-529)	571 (429-714)
	$\Delta F/F_m'$	S	<0.05	500	1000	W2.3	491 (436-545)	541 (488-595)	683 (615-750)
	Saturation	J	<0.001	250	500	LL.4	NA	NA	NA
	Polyp Retraction	J	<0.001	NA	63	LL.5	33 (27-40)	60 (52-68)	147 (136-158)
	T issue Chl-a	D	<0.05	250	500	W2.3	203 (120-286)	292 (199-385)	686 (501-871)
Test 2	Mortality	J	<0.001	250	500	LL.2	498 (477-519)	532 (450-614)	595 (344-846)
	$\Delta F/F_m'$	S	<0.05	500	1000	W2.3	497 (474-521)	536 (512-560)	638 (601-675)
	Saturation	J	<0.001	63	125	LL.5	117	166	284

							(77-156)	(127-205)	(246-322)
	Polyp Retraction	J	<0.001	NA	63	LL.5	26 (20-32)	49 (42-56)	129 (118-139)
	Tissue Chl-a	D	<0.05	250	500	W2.3	250 (162-339)	354 (263-445)	642 (541-743)
Test 3	Mortality	J	<0.001	250	500	LN.3	372 (-72-817)	410 (81-739)	494 (463-525)
	ΔF/F_m’	J	<0.001	NA	63	LL.4	414 (-136-964)	439 (39-840)	487 (383-590)
	Saturation	S	<0.001	63	125	LL.4	93 (57-130)	152 (110-194)	351 (228-474)
	Polyp Retraction	J	<0.001	NA	63	LN.3	53 (43-63)	75 (64-87)	147 (133-161)
	Tissue Chl-a	S	<0.05	125	250	W2.3	53 (6.5-99)	106 (34-177)	533 (275-791)
Average	Mortality	NA	NA	250	500	NA	449	484	553
	ΔF/F_m’	NA	NA	500	688	NA	467	505	603
	Saturation	NA	NA	125	250	NA	105	159	318
	Polyp Retraction	NA	NA	NA	63	NA	37	61	141
	Tissue Chl-a	NA	NA	208	417	NA	169	251	620

Table S12. Calculated median lethal concentrations (LC50s) for coral species of all life stages.

Reference	Species	Lifestage	Duration (h)	LC50 (mg/L)
Esquivel, 1986	*P. damicornis*	Larva	2	0.556
Esquivel, 1986	*P. damicornis*	Larva	2	0.552
Esquivel, 1986	*P. damicornis*	Larva	4	0.44
Esquivel, 1986	*P. damicornis*	Larva	4	0.438
Esquivel, 1986	*P. damicornis*	Larva	6	0.286
Esquivel, 1986	*P. damicornis*	Larva	6	0.29
Reichelt-Brushett and Harrison, 2004	*G. aspera*	Larva	6	0.248
Reichelt-Brushett and Harrison, 2004	*G. aspera*	Larva	6	0.26
Esquivel, 1986	*P. damicornis*	Larva	8	0.195
Esquivel, 1986	*P. damicornis*	Larva	8	0.19
Esquivel, 1986	*P. damicornis*	Larva	12	0.123
Esquivel, 1986	*P. damicornis*	Larva	12	0.12
Reichelt-Brushett and Harrison, 2004	*G. aspera*	Larva	24	0.121
Reichelt-Brushett and Harrison, 2004	*G. aspera*	Larva	24	0.137
Esquivel, 1986	*P. damicornis*	Larva	24	0.115
Esquivel, 1986	*P. damicornis*	Larva	24	0.114
Bao et al., 2011	*A.* tumida	Larva	24	0.08
Kwok and Ang, 2013	*P. acuta*	Larva	24	0.1033[1]
Kwok and Ang, 2013	*P. acuta*	Larva	24	0.1087[2]
Reichelt-Brushett and Harrison, 2004	*G. aspera*	Larva	48	0.04
Reichelt-Brushett and Harrison, 2004	*G. aspera*	Larva	48	0.087
Esquivel, 1986	*P. damicornis*	Larva	48	0.09
Esquivel, 1986	*P. damicornis*	Larva	48	0.087
Kwok, 2016	*P. acuta*	Larva	48	0.11
Kwok and Ang, 2013	*P. acuta*	Larva	48	0.0965[1]
Kwok and Ang, 2013	*P. acuta*	Larva	48	0.1024[2]
Reichelt-Brushett and Harrison, 2004	*G. aspera*	Larva	72	0.034
Reichelt-Brushett and Harrison, 2004	*G. aspera*	Larva	72	0.082
Esquivel, 1986	*P. damicornis*	Larva	72	0.069
Esquivel, 1986	*P. damicornis*	Larva	72	0.07

Esquivel, 1986	*P. damicornis*	Larva	96	0.057
Esquivel, 1986	*P. damicornis*	Larva	96	0.063
Kwok, 2016	*P. acuta*	Larva	96	0.107
Kwok and Ang, 2013	*P. acuta*	Larva	96	0.0933[1]
Kwok and Ang, 2013	*P. acuta*	Larva	96	0.0910[2]
Hédouin et al., 2016	*P. damicornis*	Larva	96	0.167
Hédouin et al., 2016	*P. damicornis*	Adult	96	0.175[3]
Hédouin et al., 2016	*P. damicornis*	Adult	96	0.251[4]
Brefeld et al., 2023	*M. digitata*	Adult	96	0.161
Sabdono, 2009	*G. fascicularis*	Adult	96	0.032
This Study	*G. fascicularis*	Adult	96	0.436

[1]Study conducted at 27 C; [2]Study conducted at 30 C; [3]Study conducted during summer; [4]Study conducted during winter

Supplementary Figures

Figure S1. Dose response curves for survival (mortality). These are presented as test 1 (blue), test 2 (red), and test 3 (green).

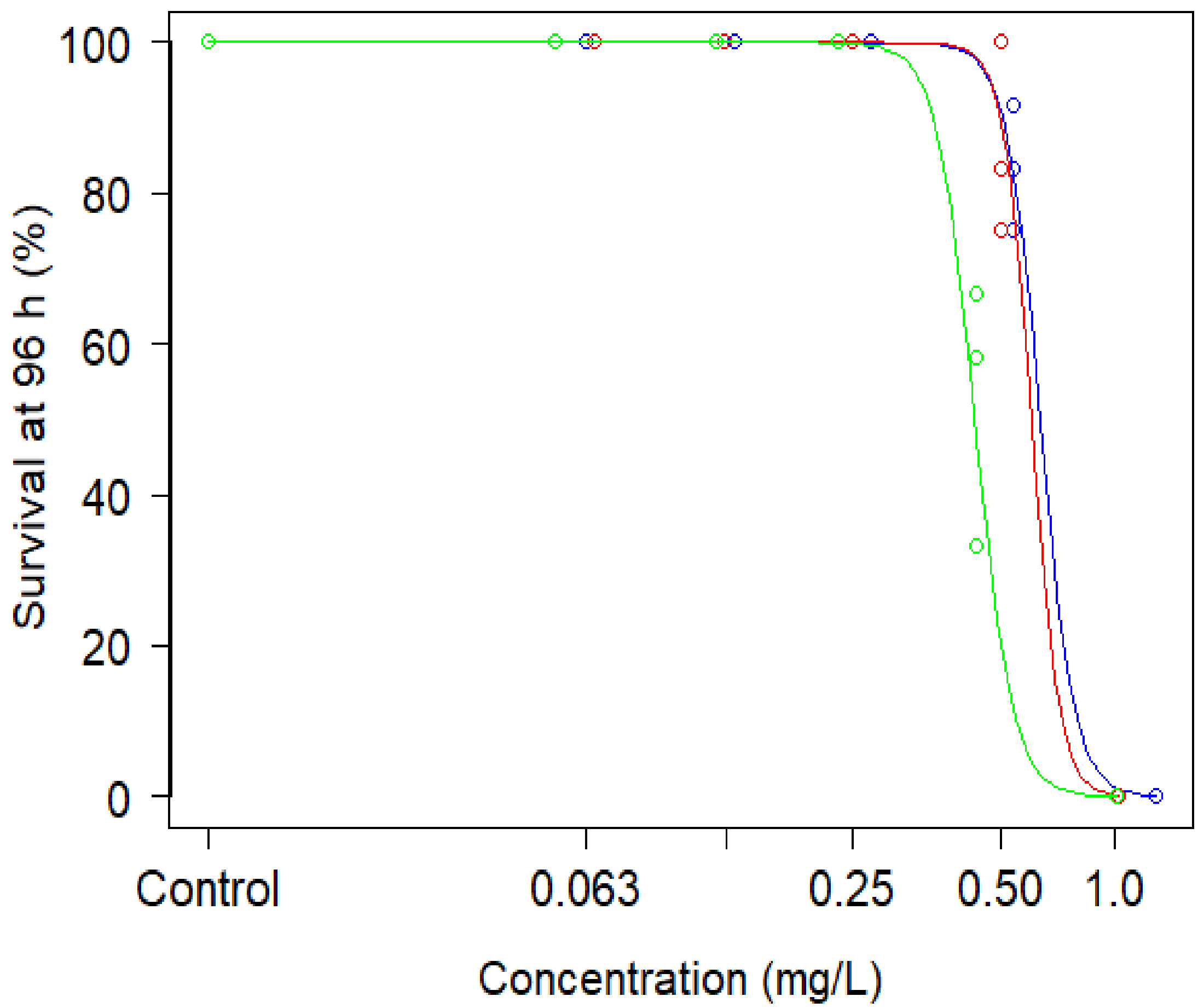

Figure S2. Dose response curves for tissue chl-*a*. These are for test 1 (blue), test 2 (red), and test 3 (green).

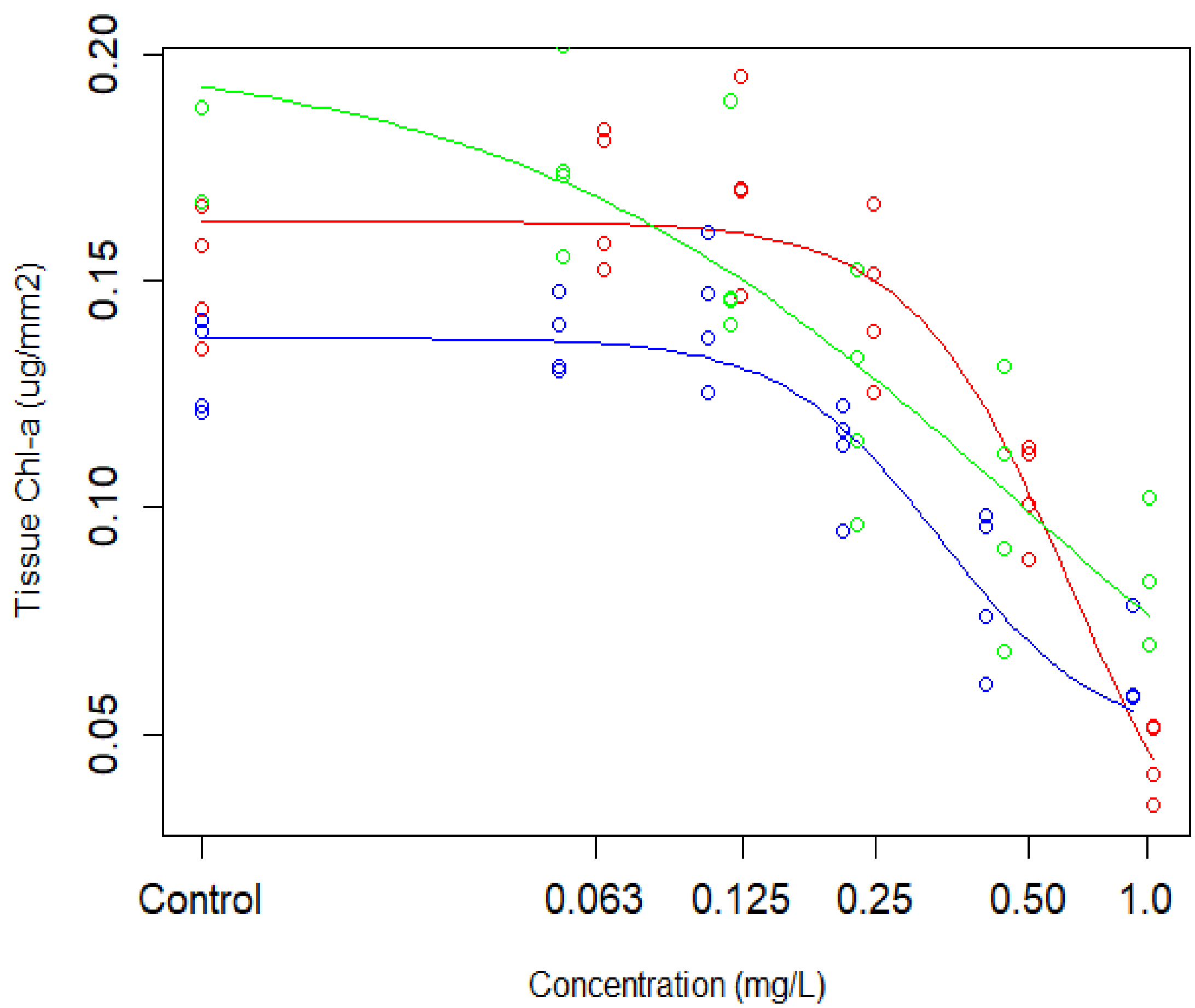

Figure S3. Dose response curves for photosynthetic efficiency. These are for test 1 (blue), test 2 (red), and test 3 (green).

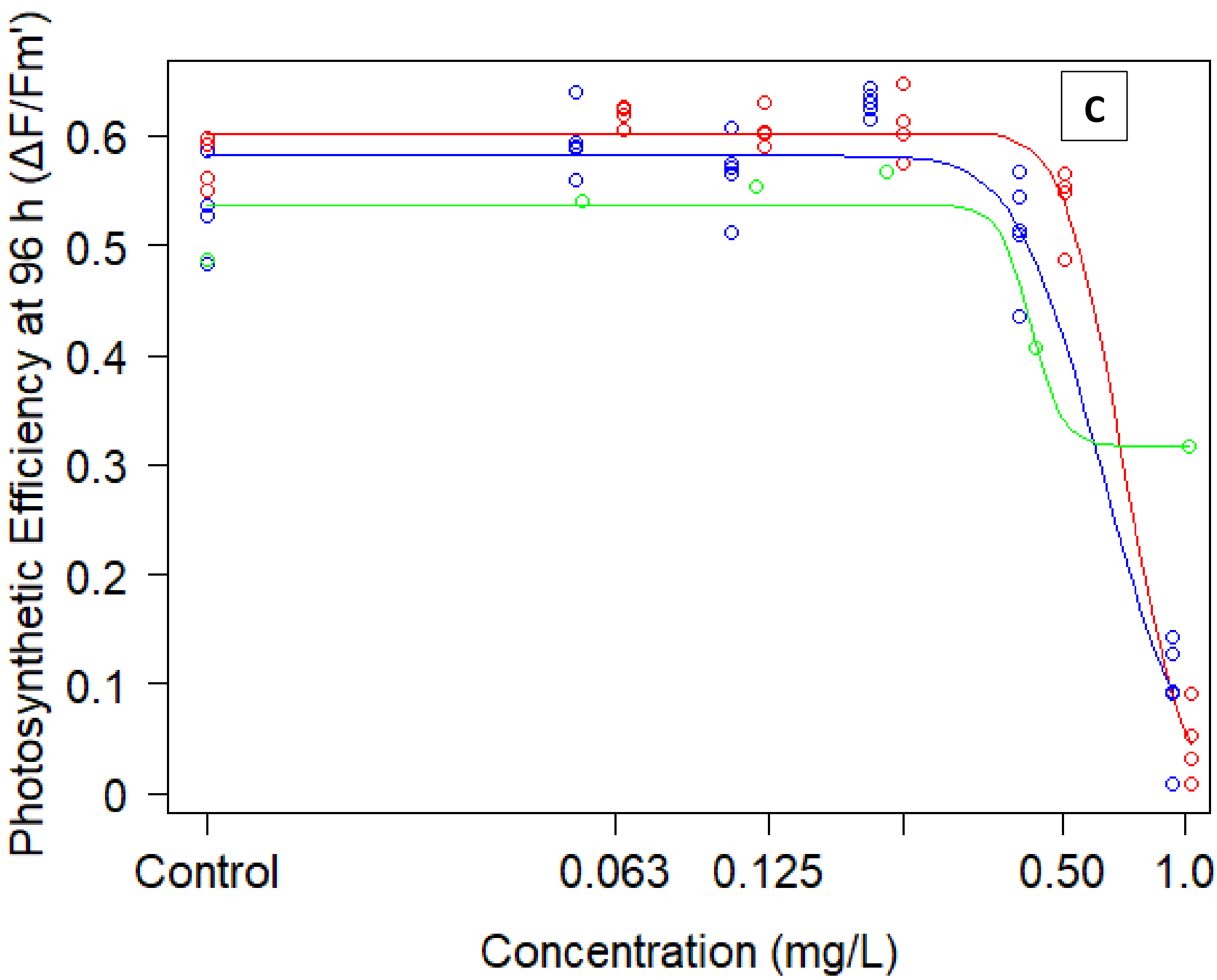

Figure S4. Dose response curves for polyp retraction. These are for test 1 (blue), test 2 (red), and test 3 (green).

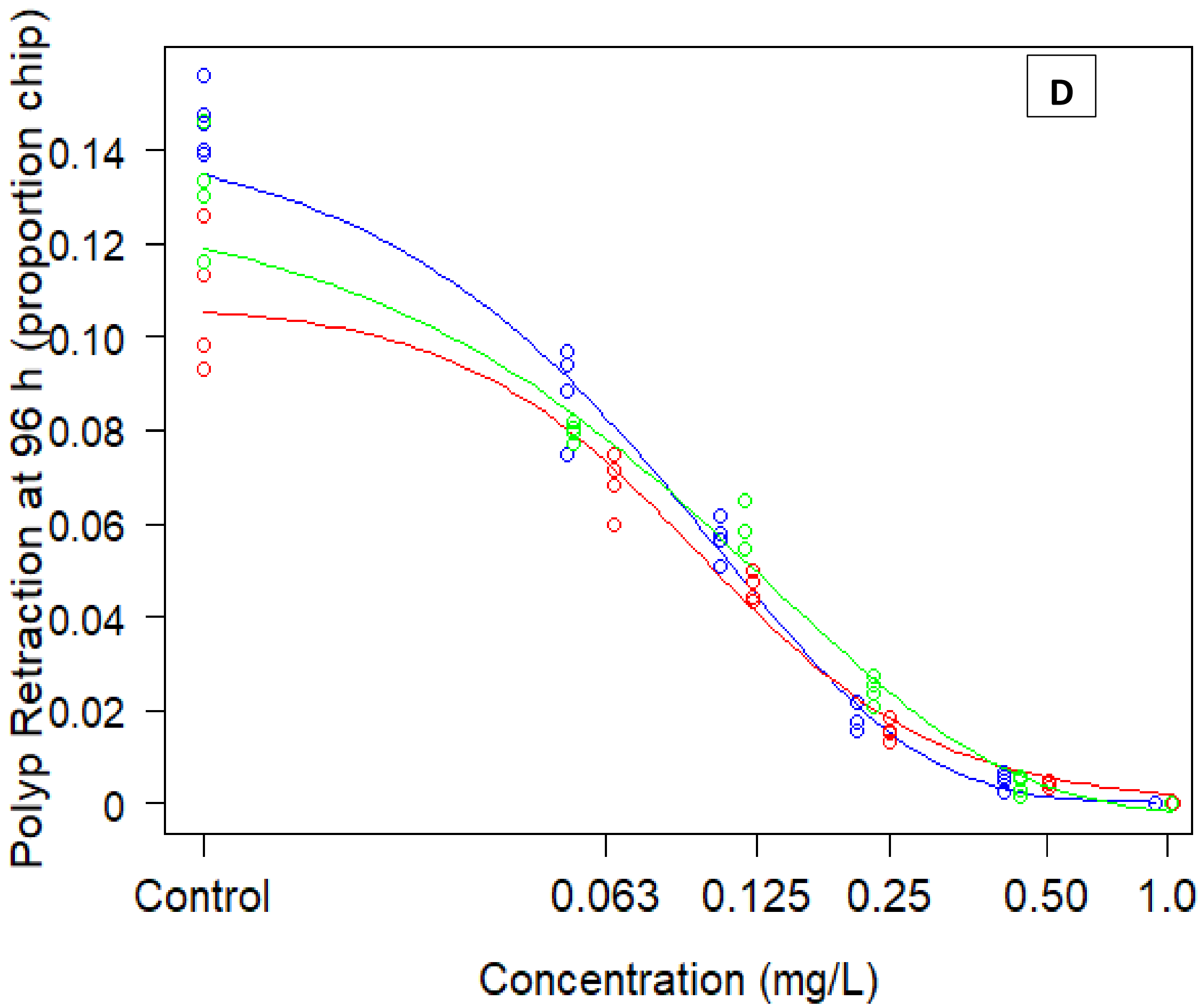

Figure S5. Dose response curves for saturation. These are for test 1 (blue), test 2 (red), and test 3 (green).

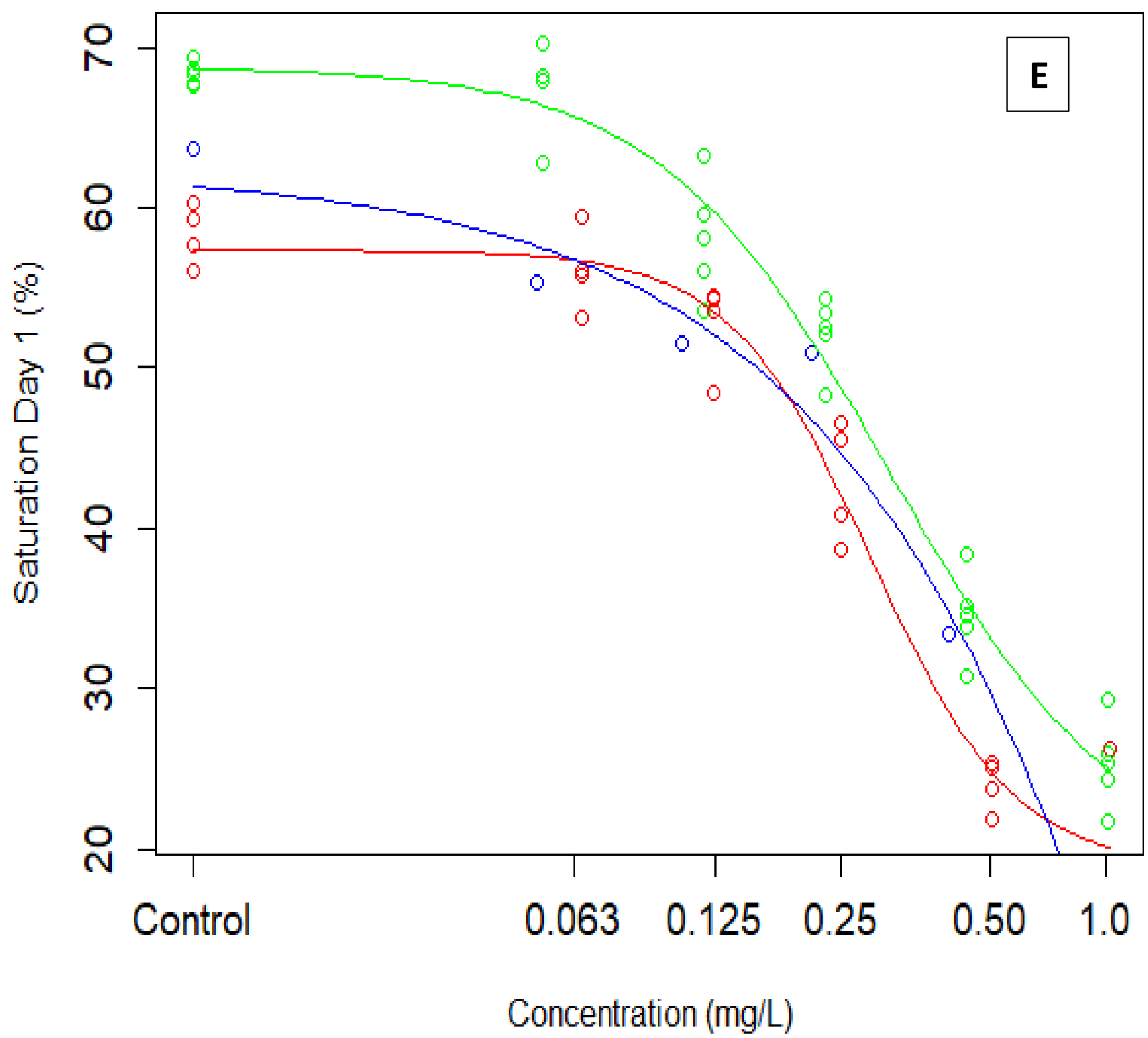

Figure S6: Chlorophyll-a concentrations in aged exposure solutions.

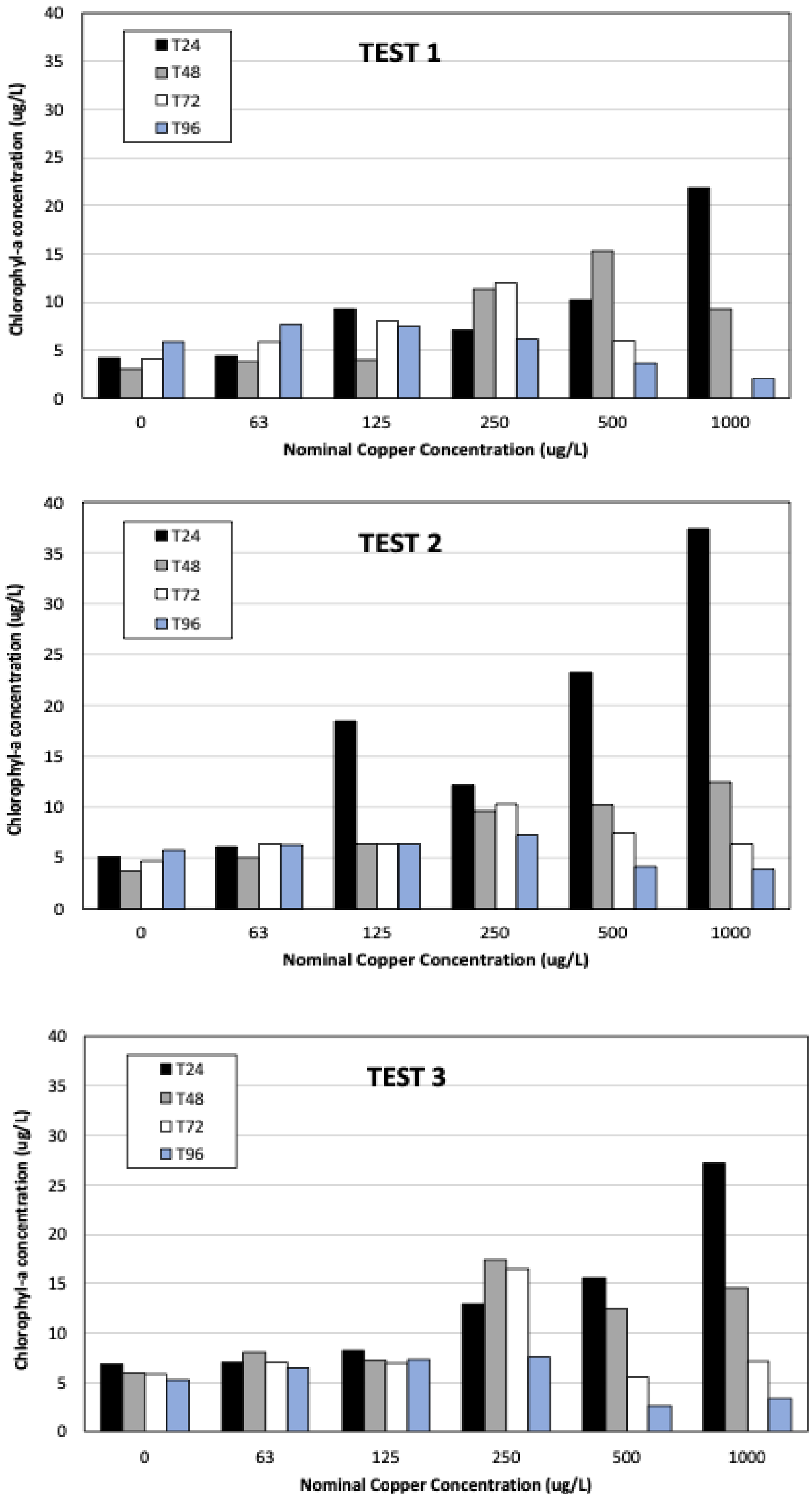

Figure S7. Species sensitivity distribution (SSD) for copper modeled using a normal and logistic model. A: SSD using copper results for 24-h median lethal concentration (LC50). B: SSD using 96-h LC50s not including the results from these tests. This generally agrees with the 24-h LC50 SSD as to the relative position of *P. damicornis* and *P. acuta*. C: SSD using 96-h LC50 with our results.

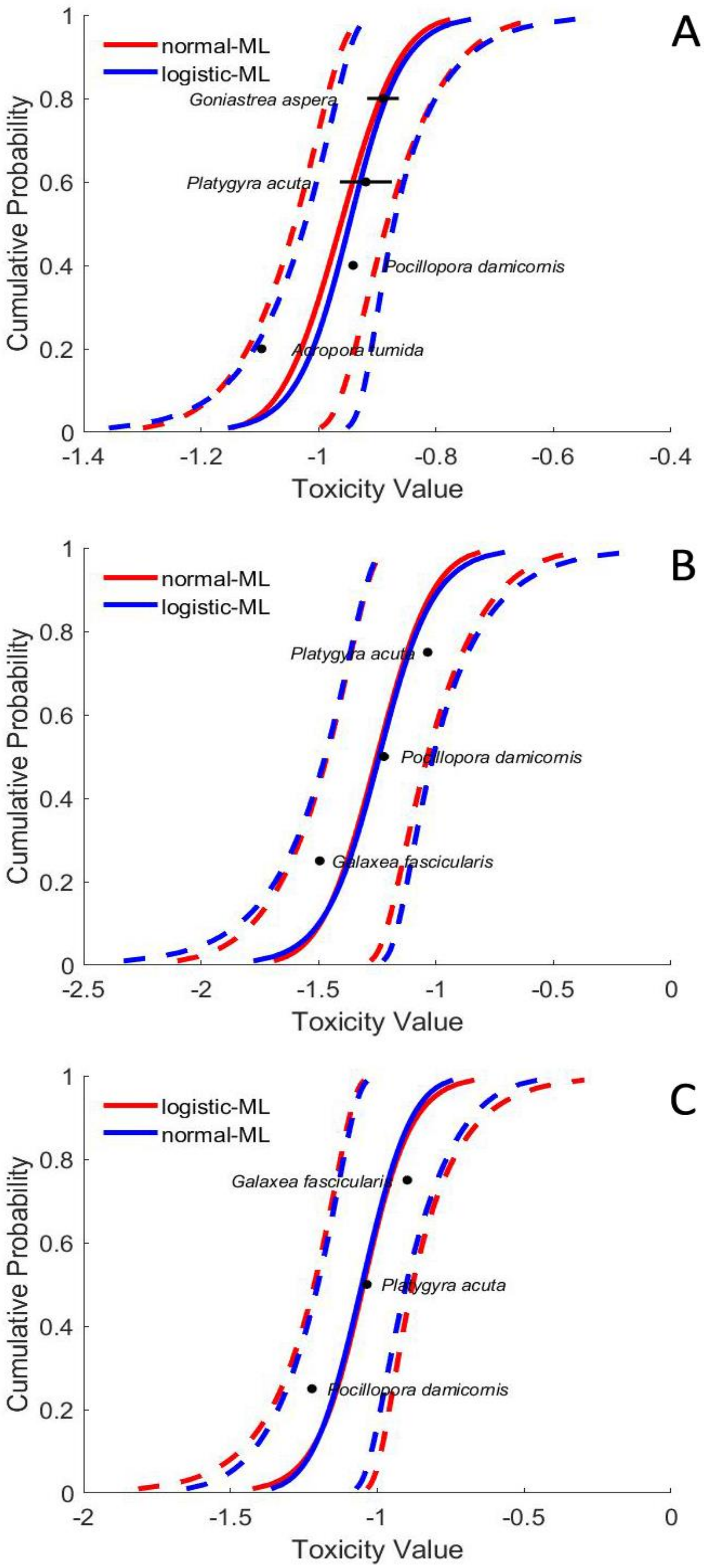
A
normal-ML
logistic-ML
Goniastrea aspera
Platygyra acuta
Pocillopora damicornis
Acropora tumida
Cumulative Probability
Toxicity Value
B
normal-ML
logistic-ML
Platygyra acuta
Pocillopora damicornis
Galaxea fascicularis
Cumulative Probability
Toxicity Value
C
logistic-ML
normal-ML
Galaxea fascicularis
Platygyra acuta
Pocillopora damicornis
Cumulative Probability
Toxicity Value

References

Conway, A.J., Gonsior, M., Clark, C., Heyes, A. and Mitchelmore, C.L., 2021. Acute toxicity of the UV filter oxybenzone to the coral Galaxea fascicularis. *Science of The Total Environment*, *796*, p.148666. https://doi.org/10.1016/j.scitotenv.2021.148666
